# Cellulose Nanocrystals as Additives in Electrospun Biocompatible Separators for Aprotic Lithium-Ion Batteries

**DOI:** 10.1021/acsapm.2c01956

**Published:** 2023-01-20

**Authors:** Antonio Laezza, Arcangelo Celeste, Mariangela Curcio, Roberto Teghil, Angela De Bonis, Sergio Brutti, Antonietta Pepe, Brigida Bochicchio

**Affiliations:** † Department of Science, University of Basilicata, Viale dell’Ateneo Lucano 10, Potenza85100, Italy; ‡ Dipartimento di Chimica, Università di Roma La Sapienza, P.le Aldo Moro 5, Roma00185, Italy; § GISEL—National Centre of Reference for Electrochemical Energy Storage Systems, INSTM, Via G. Giusti 9, Firenze50121, Italy

**Keywords:** battery separators, biocompatibility, cellulose nanocrystals, cyclic voltammetry, electrospinning, ohmic resistance

## Abstract

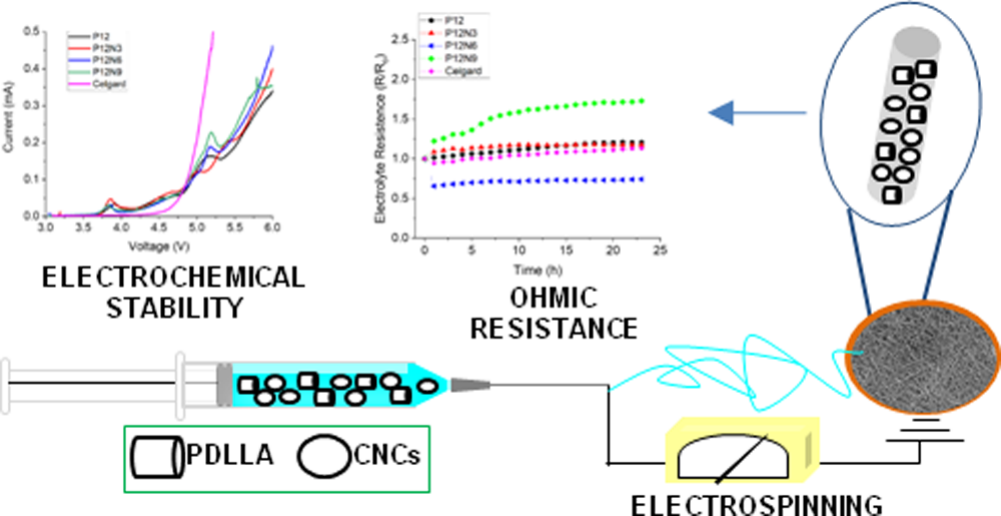

This work concerns the study of electrospun scaffolds as separators for aprotic lithium-ion batteries (LIBs) composed of the amorphous poly-d,l-lactide (PDLLA), in solution concentrations of 8, 10, and 12 wt % and in different ratios with cellulose nanocrystals (CNCs). PDLLA has been studied for the first time as a separator, taking into account its amorphous character that could facilitate electrolyte incorporation into the polymer matrix and influence ionic conductivity, together with CNCs, for reducing the hydrophobicity of the scaffolds. The embedding of the nanocrystals in the scaffolds was confirmed by X-ray diffraction analysis and attenuated total reflectance Fourier transform infrared spectroscopy. The polymer combination influenced the nanofibrous morphology as evaluated by scanning electron microscopy and modulated the electrochemical behavior of the membranes that was investigated through linear sweep voltammetry, cyclic voltammetry, and electrochemical impedance spectroscopy tests. Among the studied categories, the P12 series displayed a nonhomogeneous electrolyte resistance and electrochemical stability, differently from P10, whose results suggested their application in LIBs with standard formulation, as confirmed by a preliminary performance test of the P10N6 formulation in a full Li-ion cell configuration.

## Introduction

Portable electronic devices, together with electric vehicles, are now part of our daily lives, creating the urgent need for a safe and high-energy-density storage system.^1−3^ Lithium-ion batteries (LIBs), thanks to their high energy densities, high coulombic efficiencies, and low self-discharge features, are widely employed as power sources.^4,5^ Remarkably in 2018, LIBs provided more than 85% of the overall installed electrochemical energy storage capacity, and their demand is expected to keep growing in the next decade approximately by +300%.^6^ Consequently, the implementation of a careful control of the carbon footprint of all LIB components is inevitable together with an overall reshape of the industrial manufacturing and disposal processes, shifting from a linear to a circular paradigm.^7−9^

Typically, LIBs consist of a transition metal-layered oxide (e.g., LiCoO_2_) and graphite, respectively, as positive and negative electrodes separated by an aprotic electrolyte. Current research trends focus on enhanced substitutes for safety and cost reasons; nevertheless, unfortunately, many alternative materials settled these aspects to the detriment of performances.^10^

In the case of liquid aprotic electrolytes, this active component of the cell is typically soaked on a polymer membrane, i.e., separator, between the electrodes. The separator is a porous key component that enables electrolyte uptake and ion transport during the charge–discharge processes, avoiding internal short circuits through electrode insulation. Besides these features, it should ideally be endowed with chemical and thermal stability, high mechanical strength, thickness, and tortuosity.^11^ Generally speaking, separators have been considered passive elements in the battery, but their properties strongly affect system safety and performance. Recently, growing efforts have been tackling the development of efficient separators, ranging from methodologies of fabrication to polymer materials and polymer blends.^12^

Compared to other categories,^11,13^ nonwoven separators produced by electrospinning have been appreciated for what concerns physical and chemical issues.^14^

Electrospinning, in fact, is a powerful manufacturing method exploited in energy storage technologies and other fields,^15,16^ which generates porous polymeric nano and microfiber structures with a large surface area-to-volume ratio. Depending on different parameters, such as solution viscosity, temperature, humidity, and operating conditions,^17^ the morphology and the properties of nonwoven fiber products can be tuned and lead to the enhancement of electrochemical performances of batteries.^18,19^

Considering the composition aspect, polyethylene (PE) and polypropylene (PP) separators have been widely used due to their high mechanical strength and chemical stability. However, their low porosity and electrolyte retaining, as well as poor thermal stability and safety, represented weak points in their applications.

For this reason, the most important drivers in the research on separators deal with the overcoming of polyolefin separator drawbacks and a careful control of the environmental impact of alternatives from both the chemical composition and the manufacturing points of view.

In this sense, electrospinning turned out to be particularly helpful. Several polymeric materials have been investigated, both in combination thereof and with nanofillers as additives, for nonwoven separator development: polyvinylidene fluoride (PVDF) and its copolymers as poly(vinylidene fluoride-*co*-hexafluoropropylene) (PVDF-HFP) and poly(vinylidene fluoride-*co*-trifluoroethylene) (PVDF-TrFE)^20−25^ afforded promising results. However, separators obtained from natural and biodegradable sources are gaining attention. Polyacrylonitrile (PAN),^26,27^ polyimide (PI),^28,29^ and cellulose derivatives^30^ are just some examples of the exploring ones.

Among all others, polylactic acid (PLA) is another valuable alternative: it is a biocompatible and biodegradable polyester used in different fields, ranging from biomedical^31^ to packaging^32^ and automotive applications.^33^ Currently, only few examples of the use of PLA as LIB separators have been reported in the literature focusing on the use of pure enantiomeric forms of PLA.^34−36^

However, both the pure enantiomeric forms of PLA (PDLA and PLLA) are semicrystalline polymers,^37,38^ different from the raceme poly-d,l-lactide (PDLLA), with a lower crystalline degree, the last form being never tested in LIBs. To enhance battery performance, oxide ceramics are typically used as fillers to reduce the crystallinity of polymer separators, since this feature facilitates the electrolyte incorporation into the polymer matrix and influences ionic conductivity.^18^

However, one of the main weaknesses of polyesters as aprotic battery separators is their hydrophobicity analogous to polyolefin. On the other hand, a better control of hydrophilicity/hydrophobicity can be achieved by embedding ceramic additives within the polymer matrixes, such as cellulose nanocrystals.

In fact, the polar groups of CNCs interact with the electrolyte enhancing the uptake and resulting in an improved wettability of the separators. Equally, hydroxyls strongly interact with Li ions, redistributing them to a homogeneous deposition on the anode and suppressing lithium dendrites.^39^

Cellulose nanomaterials can be extracted by exploiting different sources and obtaining characteristics which depend on the hydrolysis conditions.^40−42^

In recent years, several studies have been reported to improve LIB performances in employing cellulosic materials from anodes^43,44^ and electrolytes^45^ to the separators.^46,47^

Moreover, besides their use for designing sustainable energy storage devices, cellulose-based polymer composites are employed in nanofibrous matrices for applications which range from water filtration^48^ to drug release.^49^

Here, we draw a comprehensive outline of a class of environmentally friendly battery separators by employing the amorphous PDLLA as a polymer host with a convenient blend of CNC fillers and investigate how their combination in different ratios can modulate the electrochemical and swelling properties, as well as the nanofibrous morphology.

## Experimental Section

### Materials and Methods

PDLLA (EasyFil PLA—PolyLactic Acid, transparent pellets, molecular weight 126,000 g/mol, density 1240 kg/m^3^) was obtained from FormFutura; cellulose nanocrystals (CNCs from wood, spray dried powder, particle size 1–50 μm, particle length 44–108 nm, particle diameter 2.3–4.5 nm, pH 6–7, 88% crystalline fraction) were bought from CelluForce, and 1,1,1,3,3,3-hexafluoro-2-propanol (HFP) was obtained from Iris Biotech GMBH. Dimethyl carbonate (DMC) and ethylene carbonate (EC) were purchased from Levanchimica SRL. Commercial-grade reagents and solvents were used without further purification, except where otherwise indicated.

### Electrospinning

All the solutions were prepared and electrospun on a Gamma High Voltage generator from Linari Engineering on a round copper plate coated with aluminum foils in the conditions listed in Table 1. In the case of neat PDLLA, they were prepared by dissolving the polymer in 3 mL of HFP and keeping them under magnetic stirring for ∼3 h at 37 °C. For the mixtures containing CNCs, CNCs were previously sieved, then dispersed in HFP, sonicated, and kept overnight under magnetic stirring at room temperature. Later PDLLA was added, and the suspension was left stirring for ∼3 h at 37 °C.

**Table 1:** Scaffold Composition

abbreviation	PDLLA % (w/V)	CNCs % (w/w)	polymer weight ratio (w/w)	final concentration % (w/V)	electrospinning process parameters			
					*V* (kV)	*N* (G)	*D* (cm)	*F* (mL/h)
P8	8			8.00	19	18	19	1.6
P8N3	8	3	1:33.3	8.24	19	18	19	1.6
P8N6	8	6	1:16.6	8.49	19	18	19	1.6
P8N9	8	9	1:11.1	8.74	19	18	19	1.6
P10	10			10.00	19	18	19	1.6
P10N3	10	3	1:33.3	10.30	19	18	19	1.6
P10N6	10	6	1:16.6	10.61	19	18	19	1.6
P10N9	10	9	1:11.1	10.93	19	18	19	1.6
P12	12			12.00	19	18	19	1.6
P12N3	12	3	1:33.3	12.36	19	18	19	1.6
P12N6	12	6	1:16.6	12.73	19	18	19	1.6
P12N9	12	9	1:11.1	13.11	19	18	19	1.6

### X-ray Diffraction (XRD) Analysis

CNC powder was analyzed by XRD and used as a standard to characterize electrospun scaffolds containing CNCs. XRD analysis was carried out on a Siemens D5000 Diffractometer. The current generator and the voltage generator were adjusted at 32 mA and 40 kV, respectively, and the step size was kept at 0.042, with a total analysis duration of 75 min.

### Attenuated Total Reflectance Fourier Transform Infrared Spectroscopy (ATR-FTIR)

ATR-FTIR spectra were recorded on electrospun scaffolds. Measurements were carried out on a Jasco J-460 instrument equipped with an ATR accessory, Smart Orbit with a type II A diamond crystal, refractive index 2.4, a KBr beam splitter, and an MCT/B detector.

Spectra were acquired in the region from 4000 to 450 cm^–1^ with a spectral resolution of 2 cm^–1^ and 32 scans. Background spectra were recorded each time and then subtracted from the sample spectra.

### Scanning Electron Microscopy (SEM)

The morphology of the electrospun scaffolds was investigated on MEB-FEG Jeol 7800F prime-EDS. After gold sputter coating, SEM images with different magnifications were acquired with a voltage of 3 kV.

Gray-scale images were manually segmented into binary black (background) and white (fiber) images using ImageJ (Java open source) software supplied with the DiameterJ plug-in (*n* > 1000). The obtained segmented images automatically allowed the determination of the distribution of fiber diameters and porosity with the same software.

Data have been reported as mean ± standard deviation. Fiber diameter and porosity were measured by using the one-way analysis of variance method and Tukey’s test. A *p*-value ≤ 0.01 (*) was considered statistically significant.

### Swelling Test Analysis

The swelling tests in 1 M LiPF_6_ EC:DMC 1:1 V/V were performed in a glovebox, after that the membranes were dried in a buchi oven. The membranes were cut into separators of 18 mm diameter and immersed in the electrolyte overnight. Five membranes of each sample were used for the analysis.

The solution and the electrolyte absorbed by each scaffold were calculated according to the following equation (eq 1):
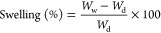
1*W*_w_ and *W*_d_ are the masses (g) of the wet and dry scaffold, respectively.

### Electrochemical Analysis

The electrochemical stability windows were evaluated by linear sweep voltammetry (LSV) for anodic scan and cyclic voltammetry (CV) for cathodic scan. SuperP carbon casted on aluminum (cathodic) or copper (anodic) foil was used as the working electrode and a lithium foil as the counter electrode. The voltage range used was 0–6 V for LSV and 0.01–2.5 V for CV, while the scan rate was 1 mV/s. Electrochemical impedance spectroscopy (EIS) tests have been performed using an Ivium Vertex instrument by applying a sinusoidal Δ*V* = 10 mV in the 100 kHz–1 Hz frequency range in the Li/Li configuration cell. Performance tests in full lithium-ion cells have been carried out by galvanostatic cycling at 30 °C at various current rates. Cells have been assembled by coupling a lithium–nickel–cobalt–aluminum layered oxide (NCA) commercial positive composite electrode (Customcells, nominal capacity 3.5 mAh cm^–2^) with a commercial negative composite electrode (active material: graphite, nominal capacity 3.8 mAh cm^–2^). The P10N6 separator has been used (see below) and swelled using a 1 M dimethyl carbonate/ethylene carbonate commercial solution (1:1 in volume, Solvionic) as the electrolyte. Galvanostatic cycling tests have been carried out using an MTI Corp battery analyzer.

## Results and Discussion

Our work aimed to design ecofriendly electrospun battery separators; therefore, the amorphous character of PDLLA for improving battery performances, along with its recyclability and biodegradability, is convenient. Rather than inorganic nanofillers, CNCs have been selected for their superior capacity to enhance composite scaffolds’ mechanical, chemical, and electrochemical properties.^50,51^

Therefore, a set of different PDLLA/CNC membranes was developed by electrospinning polymer blends described in Table 1. Polylactide was dissolved, and CNCs were dispersed in HFP and electrospun using conditions already published by our group.^52−54^

Neat PDLLA membranes (P8, P10, and P12) were obtained from solutions presenting polymer quantities ranging from 8.0 to 12.0% (w/V) in HFP. To each of them, CNCs were added after being sieved in a concentration ranging from 3 to 9% (w/w), obtaining hybrid PDLLA/CNC membranes (P8N3–P8N9, P10N3–P10N9, and P12N3–P12N9).

### XRD Analysis

**Figure 1:**
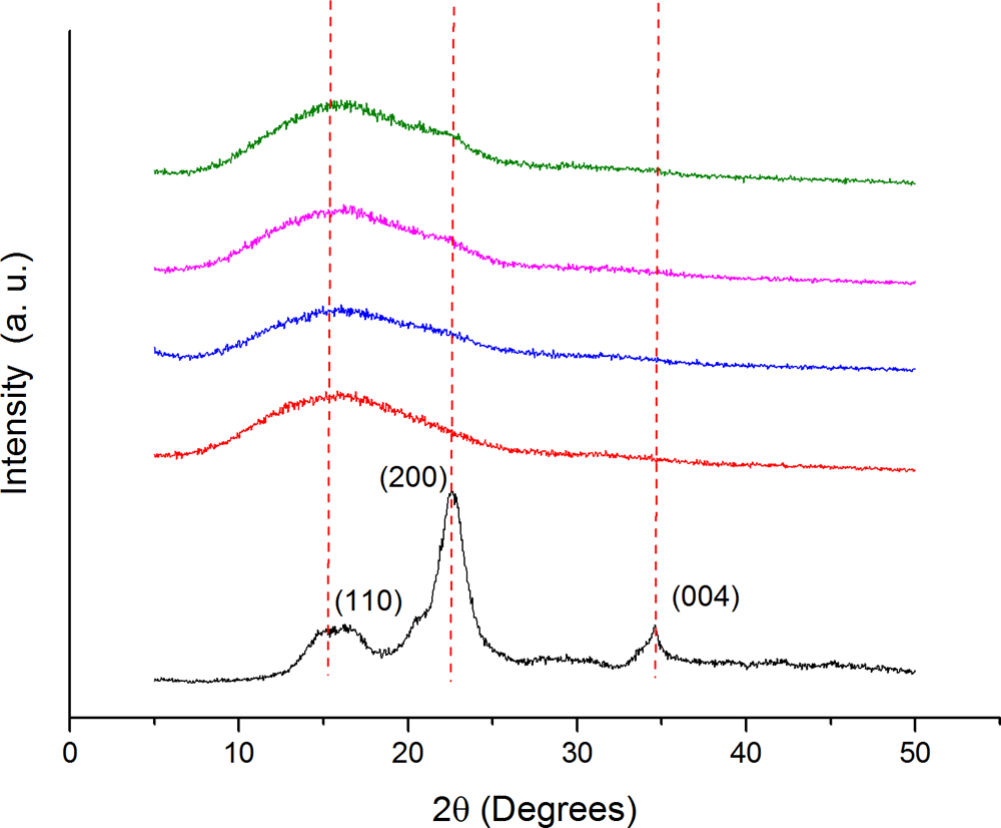
Diffractograms of electrospun scaffolds: CNC powder (black), P12 (red), P12N3 (blue), P12N6 (purple), and P12N9 (green).

For evaluating CNC’s incorporation into the hybrid scaffolds, P12 and P12N3–P12N9 hybrid electrospun scaffold diffractograms were recorded as representative behavior of all samples and compared with the CNC powder as a reference. This latter (Figure 1, black curve) showed peaks at 2θ =16.5°, 22.6°, and 34.6°, corresponding, respectively, to (1 1 0), (2 0 0), and (0 0 4) reflections of cellulose I crystallinity.^55^

Proceeding from neat PDLLA to 9% w/w CNCs containing PDLLA (Figure 1, green curve) hybrid electrospun scaffold, the shoulder at 2θ = 22.6° associated with the typical cellulose I crystalline structure was clearer, confirming that CNCs were dispersed with a higher quantity in polymer mixtures as a nanofiller, retaining their crystallinity.

### ATR-FTIR Spectroscopy

To confirm further the nanofiller presence, the hybrid membranes were characterized at a molecular level by ATR-FTIR and compared with the CNC powder as a reference (Figure S1).

Regarding CNCs (black curve), two broad bands, from ∼3600 to ∼3000 cm^–1^ and from ∼2900 to ∼2830 cm^–1^, can be attributed respectively to the O–H (filled gray circle) and to aliphatic C–H stretching vibrations (filled black circle). Typical of carbohydrates is the band at ∼1360 cm^–1^, which is related to the symmetrical C–H and O–H bending vibrations (filed red circle) the band at ∼1184 cm^–1^ associated C–O–C asymmetric stretching vibrations (filled orange circle) of β-(1 → 4) glycosidic bond in cellulose and hemicelluloses as well as the three bands at ∼1120, 1088, and 1044 cm^–1^ associated with the stretching vibrations C–CO stretching vibrations (filled brown circle).^56−58^

P12 (red curve), P12N3 (blue curve), P12N6 (purple curve), and P12N9 (green curve) showed in the region from ∼2998 to ∼2943 cm^–1^ the aliphatic C–H and C–H_3_ stretching vibrations (filled black circle) and at ∼1750 cm^–1^ the ester C=O stretching vibrations band (filled blue circle). Moreover, in the region from ∼1454 to ∼1330 cm^–1^, the spectrum displayed bands due to C–H_3_ (filled green circle) and C–H bending vibrations (filled red circle). This latter overlapped at ∼1267 cm^–1^ to ester C–O–C stretching vibrations,^59^ which generated bands at ∼1181 and at ∼1084 cm^–1^ (filled brown circle).^60^

**Figure 2:**
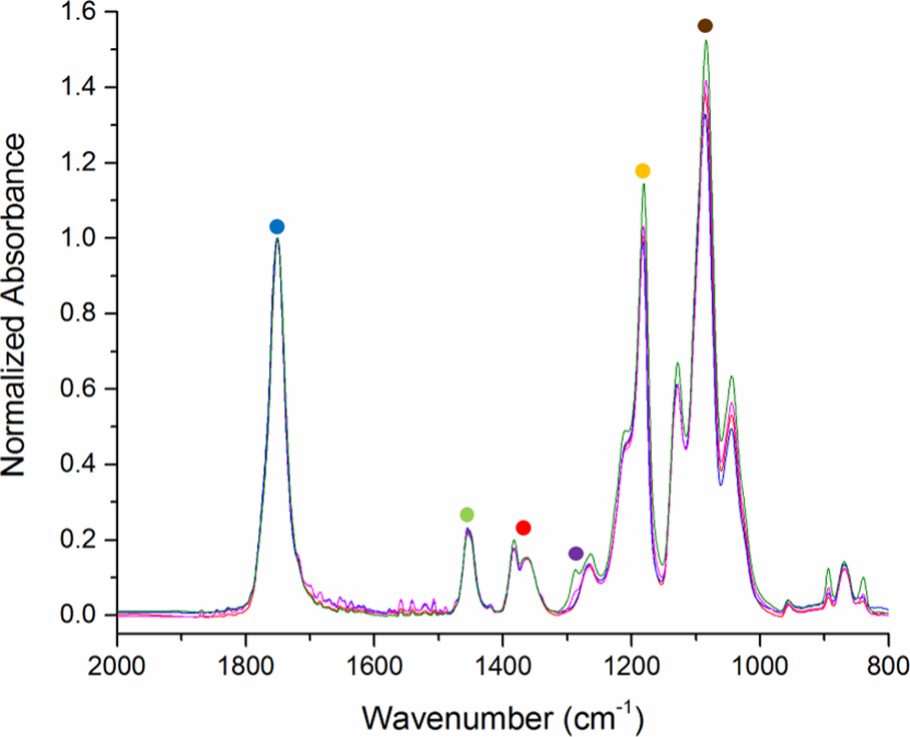
Magnification of ATR-FTIR spectra of electrospun scaffolds: P12 (red), P12N3 (blue), P12N6 (purple), and P12N9 (green).

To confirm the growing content of CNCs, the ATR-FTIR spectra of hybrid scaffolds were investigated, in the region between ∼1750 and ∼900 cm^–1^ (Figure 2), after normalization of ester C=O stretching vibrations bands (blue filled circle) from PDLLA.

In the region of interest, it can be seen that the increase of bands’ intensity at 1330 and at ∼1267 cm^–1^ associated with the polysaccharide C–H bending vibrations (filled red circle) and with polysaccharide C–H bending vibrations overlapped with C–O stretching vibrations (filled purple circle), respectively. Moreover, this feature was also confirmed by the increase of peaks in the region from ∼1200 to 1000 cm^–1^ where polysaccharide C–C and C–O symmetrical stretching fall (filled orange and brown circles).^61^

### SEM Analysis

Morphological characterization of scaffolds was conducted by SEM image analysis, focusing, besides fiber diameter, on structure uniformity and porosity since they are fundamental aspects in separator design. Both strongly influence LIBs’ performance and safety: it is well known that the absence of defects promotes the electrolyte absorption and ion transport between the electrodes and prevents short circuits in the cell thanks to the good mechanical strength of mats.^62^ Moreover, the porosity should ideally fall in the 40–60% range to ensure a balance between good ionic conductivity and mechanical strength, necessary for battery operation.^63^

**Figure 3:**
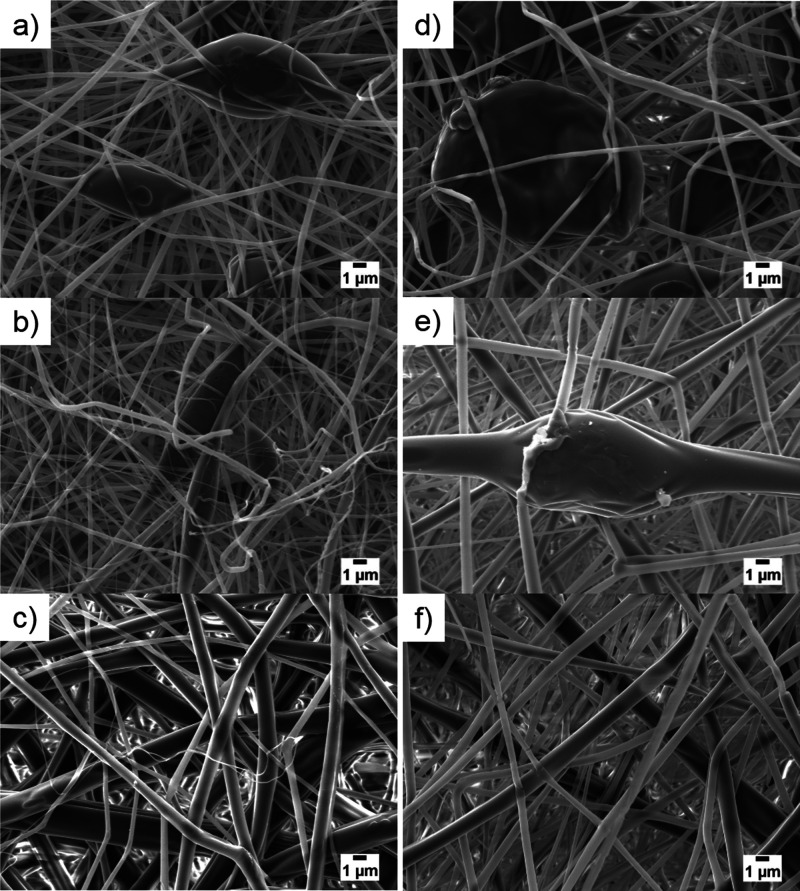
SEM images of electrospun scaffolds (a) P8, (b) P10, (c) P12, (d) P8N3, (e) P10N3, and (f) P12N3; (bar: 1 μm).

As seen in Figure 3, electrospun mats are characterized by a randomly oriented pattern of fibers. In particular, in 8 and 10% w/V PDLLA membranes, beaded morphologies were detected (Figure 3a,b), whereas, at the highest concentration of 12% w/V a uniform structure was obtained (Figure 3c). The same trend was detected in the composite separators after incorporating CNCs. P8N3 (Figure 3d), P8N6, and P8N9 (Figure S2a,d) presented beaded randomly oriented fibers, as well as P10N3 (Figure 3e), P10N6, and P10N9 (Figure S2b,e). On the contrary, defectless morphology has been detected in P12N3 (Figure 3f), P12N6, and P12N9 (Figure S2c,f).

This feature could be considered a consequence of increasing viscosity due to higher polymer concentration: a parameter responsible, in addition, for the enlargement of fibers and, consequently, the reduction of porosity.^17^

**Figure 4:**
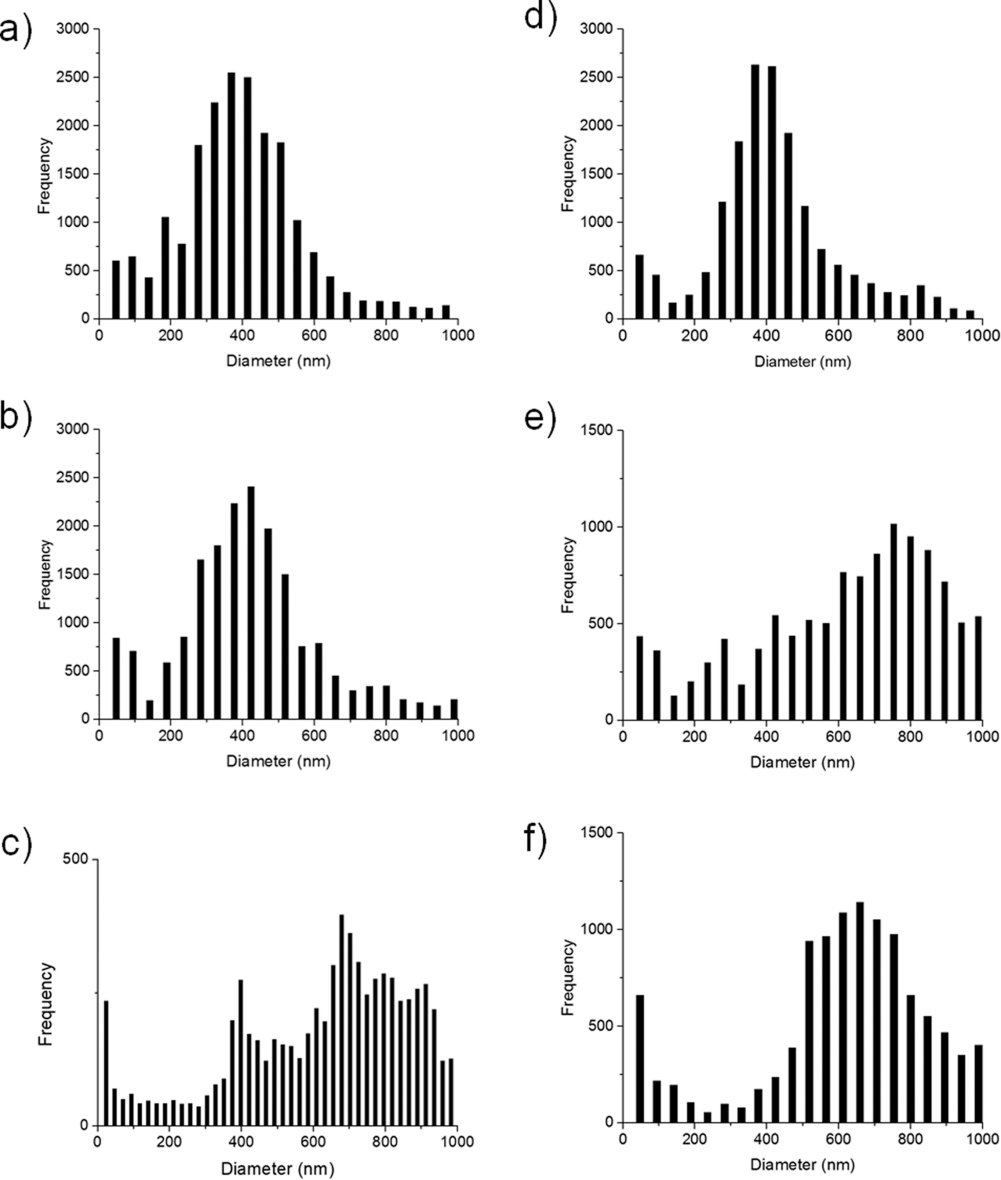
Histograms of the frequency as a function of the fibers diameter: (a) P8, (b) P10, (c) P12, (d) P8N3, (e) P10N3, and (f) P12N3.

The average diameter of fibers as a frequency function is represented as histograms (Figures 4 and S3).

Pure polylactide electrospun mats P8, P10, and P12 possessed mean diameters of 402.93 ± 21.56, 410.37 ± 11.30, and 716.10 ± 18.06 nm, respectively. Statistical analysis displayed a statistical increase (p* ≤ 0.01) of mean diameters (Figure S4a) for P12 pure polylactide mats. Nanocrystals’ addition to the electrospun mixture produced both enlargements and reductions of fibers’ mean diameter compared with neat PDLLA scaffolds, as reported in Table S1. These contrasting features are respectively associated with an increase in the viscosity and electrical conductivity of the polymer blends containing different percentages of CNCs.^53^

The addition of CNCs to 8% w/V PDLLA led to a statistically different increment (p* ≤ 0.01) of the mean diameter in the case of P8N9 (483.83 ± 21.72 nm) in comparison with P8, P8N3, and P8N6, respectively, with 402.93 ± 21.56, 393.60 ± 3.65, and 384.73 ± 12.83 nm (Figure S4b). For 10% w/V PDLLA, the addition of nanocrystals led to all statistically different (p* ≤ 0.01) samples: P10N3, P10N6, and P10N9 showed higher values in terms of mean fibers’ diameter with respect to pure P10 (410.37 ± 11.30 nm), respectively, with 746.03 ± 43.19, 617.93 ± 24.55, and 508.97 ± 4.59 nm. However, a statistical reduction has been observed with a growing quantity of nanofillers (Figure S4c).

12% w/V PDLLA nanocomposite fibers with CNCs reported at the same time statistical (p* ≤ 0.01) increments and reductions of fiber diameter both in comparison to P12 (716.10 ± 18.06 nm) and among composite scaffolds with 686.80 ± 25.12, 571.90 ± 17.66, and 898.07 ± 34.42, respectively, for P12N3, P12N6, and P12N9 (Figure S4d). These features could be ascribed simultaneously to the effect of nanocrystals on viscosity and electric conductivity of polymer blends.

Morphological analysis indicated an inverse relationship between mean diameters and scaffold porosity (Table S1): depending on data above reported, P8 and P10 displayed respectively values of 48.72 ± 3.07 and 53.50 ± 3.89%, with a statistically relevant (p* ≤ 0.01) drop to 37.11 ± 1.00 in the case of P12 (Figure S5a). Thereby, the addition of CNCs to 8% w/V PDLLA led to a reduction of porosity, moving from 3 to 9% w/w CNC-loading level, despite in P8N3 it was statistically higher (67.19 ± 4.10%) than the pure P8 scaffold and composite ones P8N6 and P8N9, respectively, with 48.72 ± 3.07, 52.74 ± 2.35, and 39.61 ± 6.30% (Figure S5b). Differently, in 10% PDLLA-containing scaffolds, the inclusion of additives first provoked a drop in porosity compared to P10 (53.50 ± 3.89%). Then their crescent quantity induced a rise with P10N3 (34.29 ± 4.89%) lower than P10N6 (41.42 ± 4.69%) and P10N9 (52.26 ± 5.01%). Statistical analysis showed a relevant difference only among P10, P10N3, and P10N9 (Figure S5c). The effect of nanocrystals for 12% w/V PDLLA scaffolds was a gradual increment in porosity compared to P12 (37.11 ± 1.00%), followed by a decline: P12N3, P12N6, and P12N9 exhibited, respectively, values of 50.18 ± 3.26, 53.99 ± 1.50, and 45.19 ± 3.23%, with a statistical relevance among P12, P12N3, and P12N6 (Figure S5d).

Membranes with the best morphological features were obtained with 12% w/V PDLLA mixtures and, for this reason selected, for further studies, using 10% w/V of poly-(d,l)-lactide ones as a term of comparison, whereas 8% w/V PDLLA and relative nanocomposite scaffolds were discarded due to a large number of beads in the fibers.

### Swelling Test

The porosity values close to the optimal range and the hydrophilicity of the embedded CNCs are expected to guarantee good wettability of the electrospun membranes.

Wettability is a relevant parameter for evaluating separators’ impact on ionic conductivity and cell performance since inadequate wetting is responsible for the internal resistance to Li^+^ ion transport and dendrite growth.

**Figure 5:**
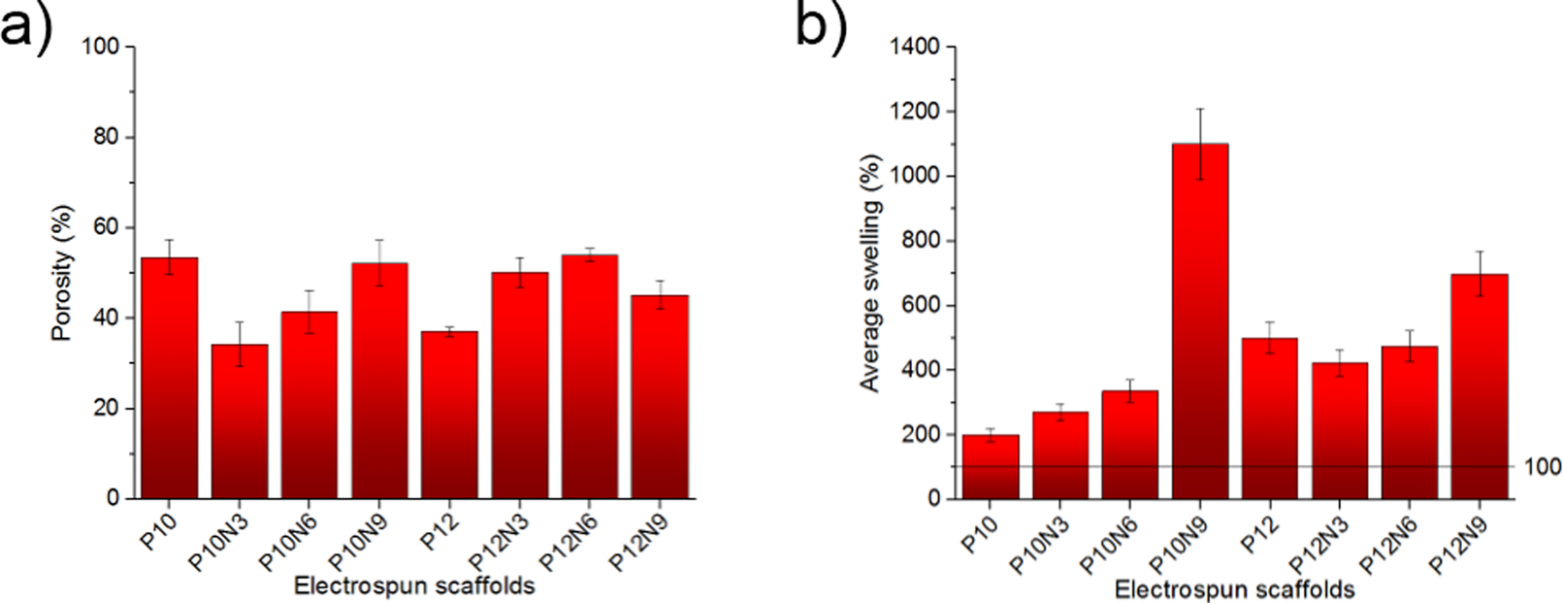
Histograms of scaffolds: (a) porosity and (b) swelling test in 1 M LiPF_6_ EC:DMC 1:1 V/V solution. Error bars represent standard deviation.

The wettability was evaluated by soaking membranes in a commercial electrolyte for LIBs. A 1 M LiPF_6_ EC:DMC 1:1 V/V electrolyte solution has been used to soak the scaffolds in an argon-filled glovebox. Results, obtained by applying the eq 1, are summarized in Table S1 and Figure 5b. Despite the porosity trend in P10-based scaffolds (Figure 5a), the swelling remains slightly below those obtained for P12-based ones, except for P10N9 (Figure 5b).

The additive-free P10 membrane shows an increase of 201 ± 20% in weight by swelling in the electrolyte: apparently the wettability grows remarkably with the addition of CNCs, increasing to 270 ± 27, 336 ± 34, and 1100 ± 110% respectively for P10N3, P10N6, and P10N9. A very close trend is also observed for P12 series. In fact, besides the small drop in the electrolyte uptake in P12N3 (423 ± 42%) compared to the pure P12 (500 ± 50%), the increase in the doping by CNCs corresponds to an increase in the electrolyte uptake.

Overall, all uptakes measured by swelling the membranes with the electrolyte are due to the strong affinity of CNC moieties with ions.

### Electrochemical Analysis

**Figure 6:**
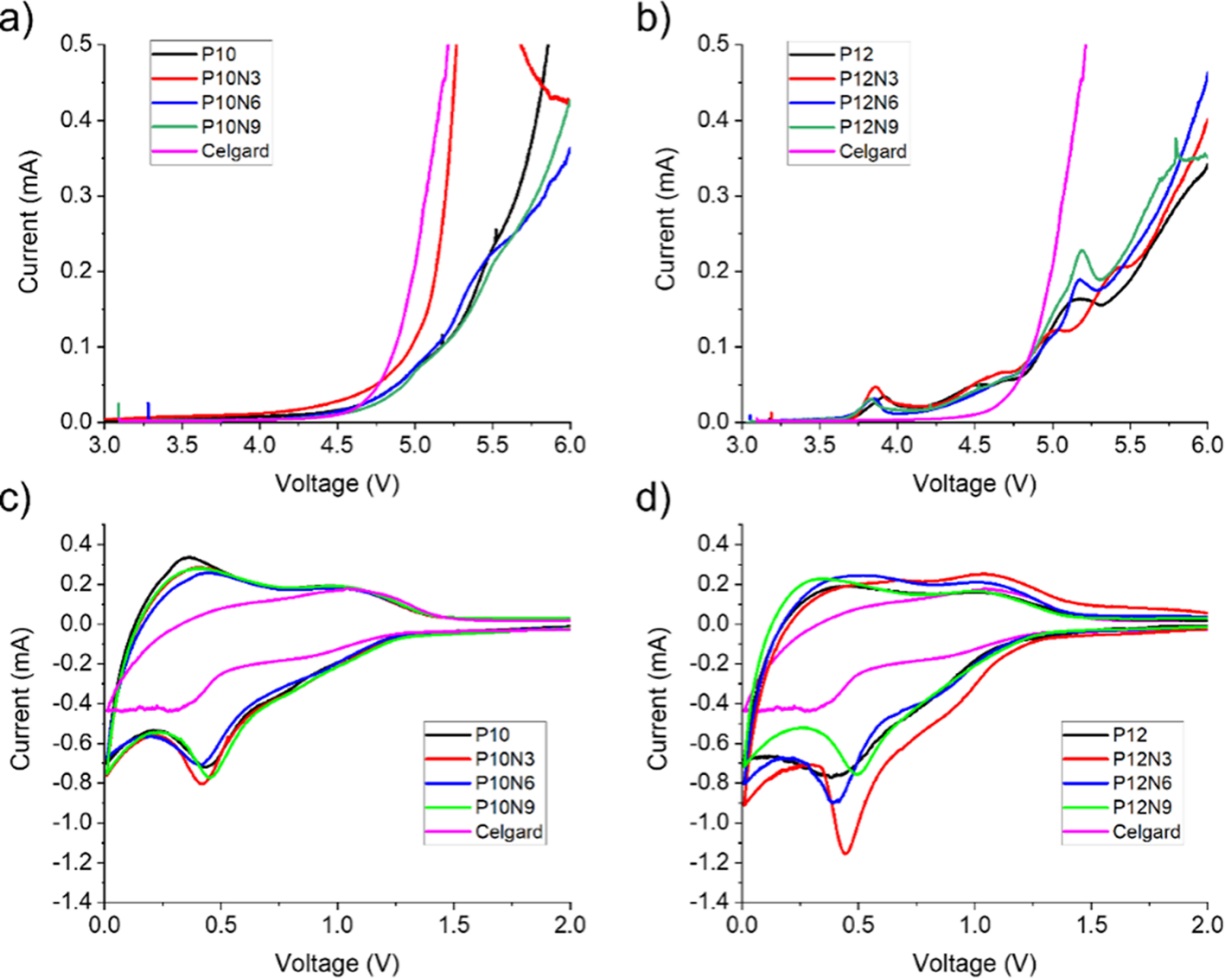
LSV of (a) P10 and (b) P12 series measured in the voltage range between 3 and 6 V at a scan rate of 1 mV s^–1^. CV first cycles of (c) P10 and (d) P12 series acquired in the voltage range of 0.01–2 V at a scan rate of 1 mV s^–1^. Celgard is reported as a comparison.

In order to measure the electrochemical stability window of the scaffolds, LSV and CV tests have been carried out.^64^ SuperP carbon electrodes casted on copper or aluminum were used as working electrodes, while Li foils as counter electrodes and LP30 as the electrolyte. Celgard separators were used as a comparison for all electrochemical tests. Figure 6 shows the obtained results for the anodic (LSV) and the cathodic (CV) scans.

Focusing on the anodic scan (Figure 6a), the P10 series showed voltage onsets of the irreversible degradation of the electrolyte at about 4.5–4.6 V vs Li, except for P10N3. These anodic thresholds closely match the Celgard separator benchmark performance. On the other hand, the P12 series shows a different behavior (see Figure 6b); in fact, it is possible to see several anodic predecomposition peaks at about 3.8 V vs Li, followed by a current drift at higher voltages. In this case, all samples show deteriorated performance compared to Celgard, being the degradation onset and kinetics worsened in all cases.

Turning to the cathodic branch, Figure 6c,d shows the first cycles of cyclic voltammetry, respectively for the P10 and P12 series, while the second and the third cycles are reported in Figures S6 and S7. The expected irreversible feature around 0.5 V vs Li^+^ is observed in both series of membranes in the first cathodic polarization and, it disappears in the subsequent cycles. This peak originates from the inevitable reduction of the electrolyte solvent species to form the stable solid electrolyte interface (SEI) over the carbonaceous components of the working electrode. The stability of the formed SEI is demonstrated by the disappearance of the 0.5 V vs Li peak: in fact, in the second and the third cycles, only the peaks of the lithium intercalation in the SuperP carbon are visible.^65^ Apparently, all scaffolds show very similar electrochemical activity in the CV tests without remarkable effects originated by the addition of CNCs.

Starting from LSV and CV data, the electrochemical stability windows (ESWs) of the different membranes can be estimated (see Table 2).

**Table 2:** Electrochemical Stability Window of the Electrospun Membranes

abbreviation	ESW V vs Li	abbreviation	ESW V vs Li
Celgard	0–4.72		
P10	0–4.6	P12	0–4.0
P10N3	0–4.5	P12N3	0–4.0
P10N6	0–4.6	P12N6	0–4.0
P10N9	0–4.6	P12N9	0–4.0

Overall, our estimates of the ESW suggests a straightforward applicability of the P10 series in LIBs with standard formulation (e.g., graphite and layered oxides) whereas the P12 series requires the use of a low-voltage positive electrode, like LiFePO_4_, due to the smaller onset anodic limit.

Besides ESWs, the chemical stability of separators toward lithium is another relevant performance parameter for LIB applications.^66,67^ In this respect, the impedance responses of symmetric Li/Li cells have been measured for 24 h at a temperature of 30 °C. The spectra for all samples and Celgard separator are shown in Figure S8. Starting for the EIS spectra, it is possible to evaluate the cell ohmic resistance (or electrolyte resistance) that is mainly related to the ionic transport across the separator,^68^ as well as the charge transfer resistance (or interface resistance), that is due to the deposition/stripping of lithium to/from the metallic surfaces.

**Figure 7:**
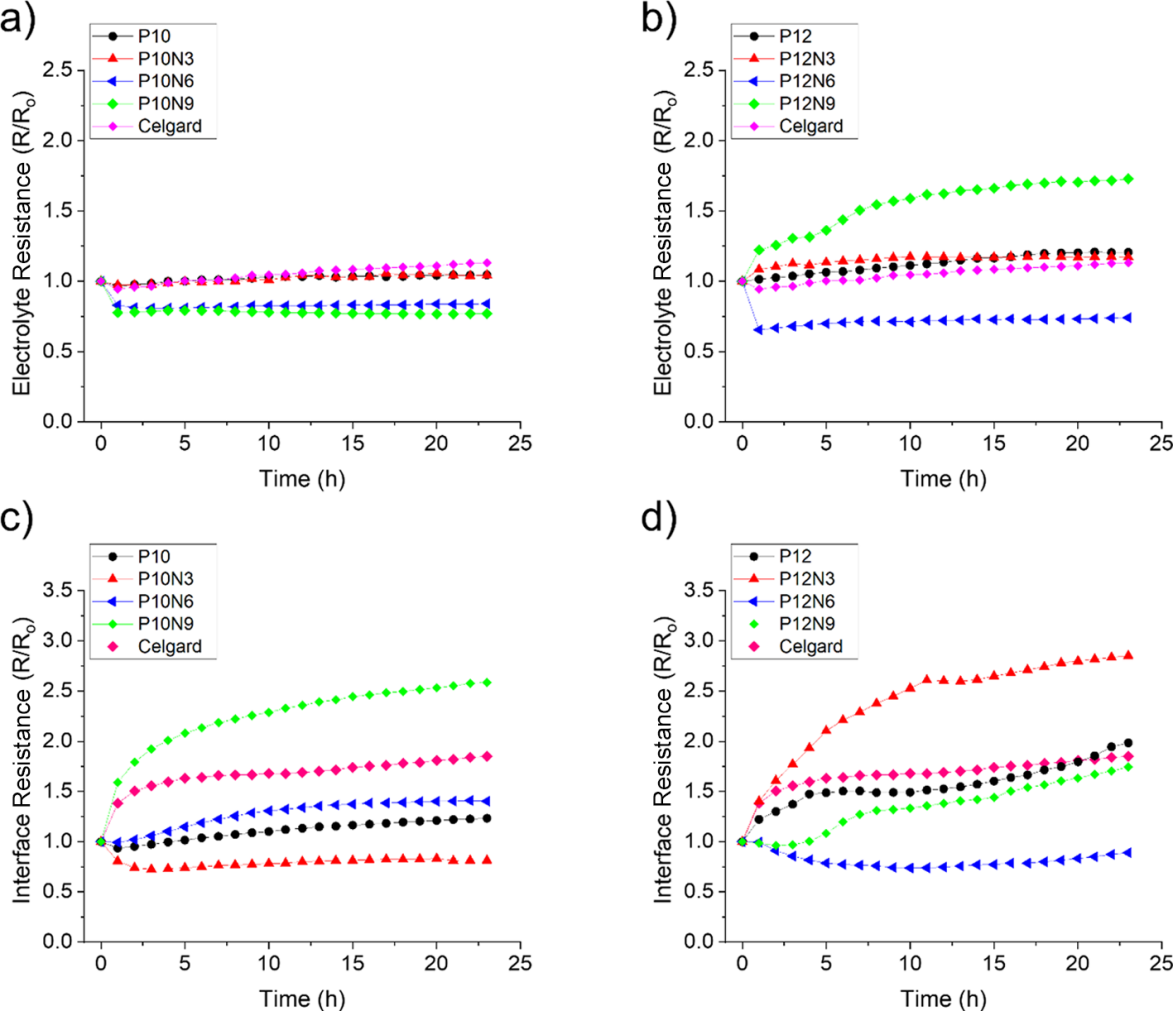
(a–d) Electrolyte resistance and interface resistance calculated from EIS spectra for the synthesized scaffolds. Celgard was added as a comparison.

The ohmic resistance as well as the charge transfer resistance is constant in an ideal Li/Li symmetric cell, and therefore, their monitoring is useful to highlight the occurrence of spontaneous chemical parasitic reactions between lithium, electrolyte, and separator that may impact the motion of ions in the electrolyte and the electrokinetics of the plating/stripping. The obtained resistances as a function of stored hours are reported in Figure 7a,b, respectively for P10 and P12 series.

The additive-free P10 membrane shows a remarkably constant electrolyte resistance upon time, very close to the commercial Celgard. The addition of the CNC additive leads in variations of the electrolyte resistance evolution upon time: it is apparently quite similar for the P10N3 scaffold, whereas it shows smaller relative values compared for the P10N6 and the P10N9 ones. In these last two cases, this trend may suggest a decrease in the tortuosity with the addition of CNCs.

Turning to the P12 series, the behavior of samples is less homogeneous. Only P12N6 shows an evolution of the electrolyte resistance better than the Celgard separator, whereas P12 and P12N3 have resistance values close to the commercial separator. P12N9 suffers a sudden huge increase in the electrolyte resistance even only few hours of direct contact with lithium. Furthermore, stable electrolyte resistance values upon time are observed only for the P12N6 membrane.

On the other hand, Figure 7c,d reports the interface resistance evolution mainly originating from the charge transfer resistance. Regarding P10 series, P10, P10N3, and P10N6 showed a less relative resistance compared to the Celgard, while P10N9 has an increase during the whole test. A more complex situation was found for P12 family where only P12N6 shows stable resistance during the test. All other samples have a huge increase in the resistance or at least a worst behavior than the Celgard.

Overall, the electrochemical stability tests suggest that only the P10, P10N6, and P12N6 membranes are suitable as separators for lithium metal-based cell applications whereas in all other cases, unstable interfaces form leading to large and scattered values of the lithium/(electrolyte & separator) interface resistances.

Among all the tested separators, the P10N6 formulation has been selected for further preliminary electrochemical tests in a full Li-ion cell configuration.

**Figure 8:**
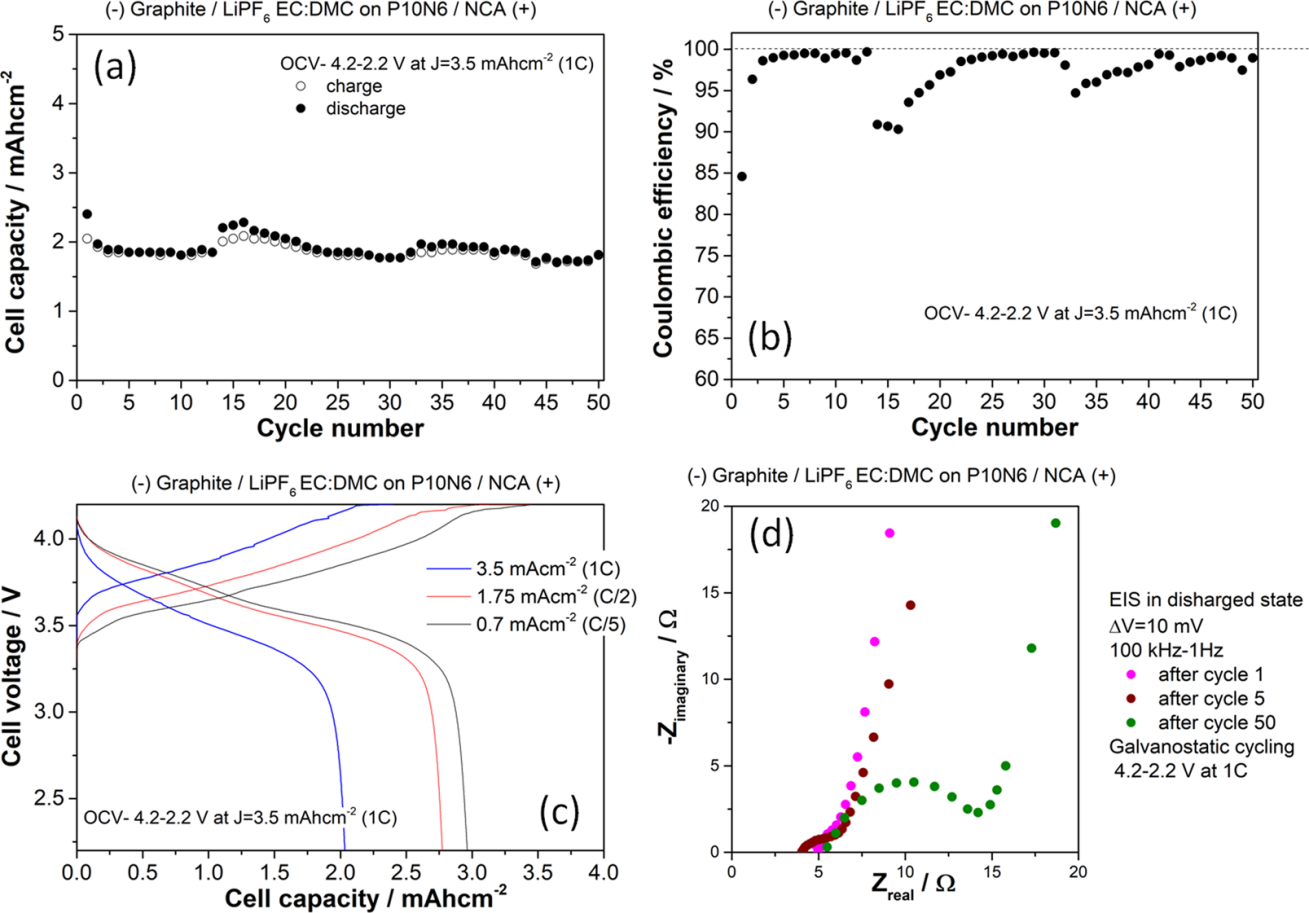
Performance of a full Li-ion NCA/Graphite cell assembled using the P10N6 separator. (a) Charge/discharge capacities at 1C; (b) coulombic efficiencies recorded upon charge/discharge at 1C; (c) cell voltage profiles at three different current rates; (d) evolution of the EIS spectra recorded upon cycling at 1C in the discharge state.

Commercial composite positive and negative electrodes, namely NCA and graphite (nominal capacity 3.5 and 3.8 mAh cm^–2^) and a commercial electrolyte (1 M LiPF_6_ EC:DMC 1:1 v/v) have been used in order to check the applicability of the P10N6 separator. Performance test results are shown in Figure 8. The separator is able to deliver very promising reversible performance: the NCA/graphite cell supplies 85, 80, and 59% of the nominal capacity at C/5, C/2, and 1C, respectively, (see Figure 8c) with a capacity retention >95% after 50 cycles at 1C. The coulombic efficiency is >98% at cycle 50 shows some reversibility drops in the first 30–35 cycles, possibly suggesting the occurrence of a limited degradation of the electrolyte/separator. This behavior also reflects on the bulk resistance of the cell estimated by the EIS experiments upon cycling (see Figure 7d) that increases by approximately 15% in the first 50 cycles. Thus, despite this minor drawback that can be addressed by an appropriate formation procedure, the performance test confirmed the applicability of the P10N6 separator in a full Li-ion cell.

## Conclusions

In the present work, a set of electrospun membranes to be employed as separators in aprotic LIBs has been developed with different amounts of PDLLA and CNC fillers. The morphology of the nanofibrous membranes improved proportionally to the quantity of polymers.

Among the evaluated separators, the P10 series exhibited the most encouraging results, with neat P10 and P10N6. They displayed homogeneous electrolyte resistance and electrochemical stability, and their electrolyte uptake was empowered gradually with the CNCs embedding empowered electrolyte uptake, gradually with the nanofiller content. For a practical evaluation of a hybrid scaffold, once assembled in a full Li-ion cell configuration using commercial electrodes and electrolytes, the P10N6 separator successfully delivered performance in terms of reversibility and rate response. These findings all suggest the applicability of the PDLLA-based separators for lithium-based cells.

## Supporting Information Available

ATR-FTIR spectra, SEM images, fiber diameter distribution histograms, fiber diameter histograms, porosity histograms, morphology and wettability data, cyclic voltammetry curves, and electrochemical impedance spectroscopy curves (PDF)
