# Supplementary material for: Cellulose Nanocrystals as Additives in Electrospun Biocompatible Separators for Aprotic Lithium-Ion Batteries
Source: ACS Appl Polym Mater. 2023 Jan 20;5(2):1453–63. doi: 10.1021/acsapm.2c01956 (PMC9926463; doi:10.1021/acsapm.2c01956)
Supplement: Supplementary file 1 — ap2c01956_si_001.pdf [file ap2c01956_si_001.pdf]

Supporting Information

Cellulose nanocrystals as additives in electrospun biocompatible separators for aprotic lithium-ion batteries

Antonio Laezza[a]*, Arcangelo Celeste[b], Mariangela Curcio[a], Roberto Teghil[a], Angela De Bonis[a], Sergio Brutti[b,c], Antonietta Pepe[a], Brigida Bochicchio[a]

[a] Department of Science, University of Basilicata, Viale dell'Ateneo Lucano 10, 85100 Potenza (IT)

[b] Dipartimento di Chimica, Università di Roma La Sapienza, P.le Aldo Moro 5, 00185 Roma (IT)

[c] GISEL—National Centre of Reference for Electrochemical Energy Storage Systems, INSTM, Via G. Giusti 9, 50121 Firenze (IT)

(A. A)* Email: antonio.laezza@unibas.it

Table of content

Figure S1: ATR-FTIR spectra of electrospun scaffolds: CNCs powder (black), P12 (red), P12N3 (blue), P12N6 (purple), P12N9 (green).	**S3**
Figure S2: SEM images of electrospun scaffolds: P8N6 (A), P10N6 (B), P12N6 (C), P8N9 (D), P10N9 (E), and P12N9 (F)	**S4**
Figure S3: Histograms of the frequency as a function of the fibers diameter: P8N6 (A), P10N6 (B), P12N6 (C), P8N9 (D), P10N9 (E), P12N9 (F)	**S5**
Figure S4 A: Fiber diameter histogram of electrospun scaffolds P8, P10, and P12	**S6**
Figure S4 B: Fiber diameter histogram of electrospun scaffolds P8, P8N3, P8N6, and P8N9	**S7**
Figure S4 C: Fiber diameter histogram of electrospun scaffolds P10, P10N3, P10N6, and P10N9	**S8**
Figure S4 D: Fiber diameter histogram of electrospun scaffolds P12, P12N3, P12N6, and P12N9	**S9**
Figure S5 A: Porosity histogram of electrospun scaffolds P8, P10, and P12	**S10**
Figure S5 B: Porosity histogram of electrospun scaffolds P8, P8N3, P8N6, and P8N9	**S11**
Figure S5 C: Porosity histogram of electrospun scaffolds P10, P10N3, P10N6, and P10N9	**S12**
Figure S5 D: Porosity histogram of electrospun scaffolds P10, P10N3, P10N6, and P10N9	**S13**
Table S1: Scaffolds morphology and wettability	**S14**
FigureS6: Cyclic Voltammetries of electrospun scaffolds P10, P10N3, P10N6, P10N9, and Celgard.	**S17**
FigureS7: Cyclic Voltammetries of electrospun scaffolds P12, P12N3, P12N6, P12N9, and Celgard.	**S18**
FigureS8: Electrochemical impedance spectroscopy of electrospun scaffolds P10, P10N3, P10N6, P10N9, P12, P12N3, P12N6, P12N9 and Celgard	**S19**

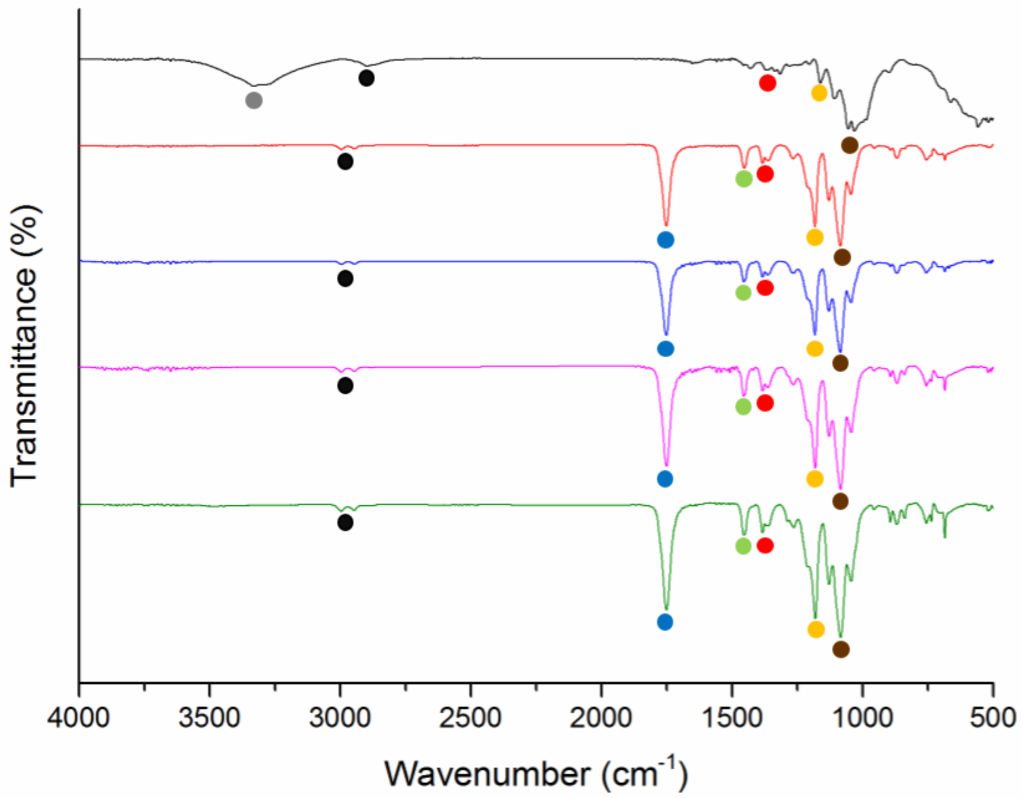

Figure S1: ATR-FTIR spectra of electrospun scaffolds: CNCs powder (black), P12 (red), P12N3 (blue), P12N6 (purple), P12N9 (green).

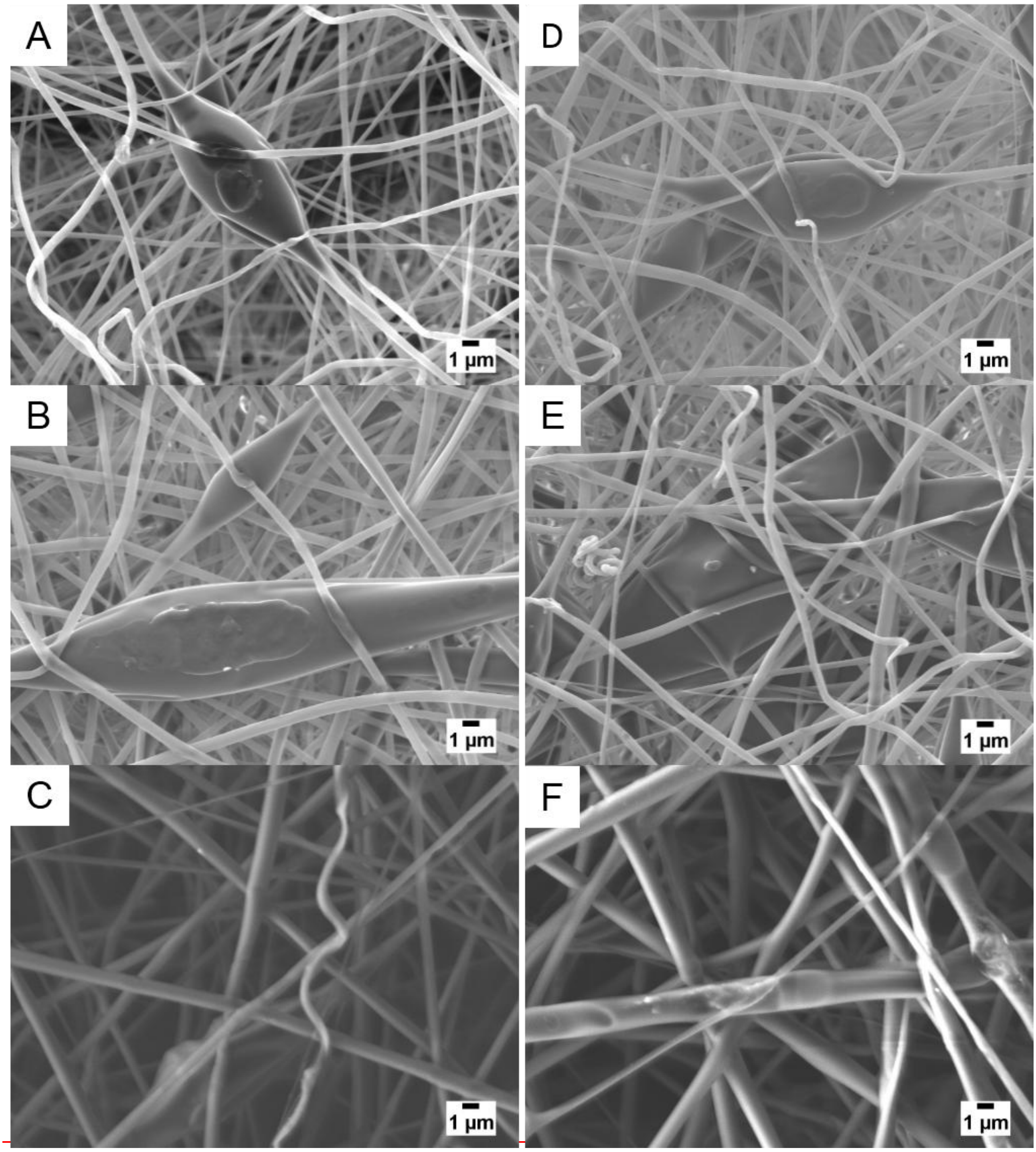

Figure S2: SEM images of electrospun scaffolds. P8N6 (A), P10N6 (B), P12N6 (C), P8N9 (D), P10N9 (E), and P12N9 (F); (bar: 1 μm).

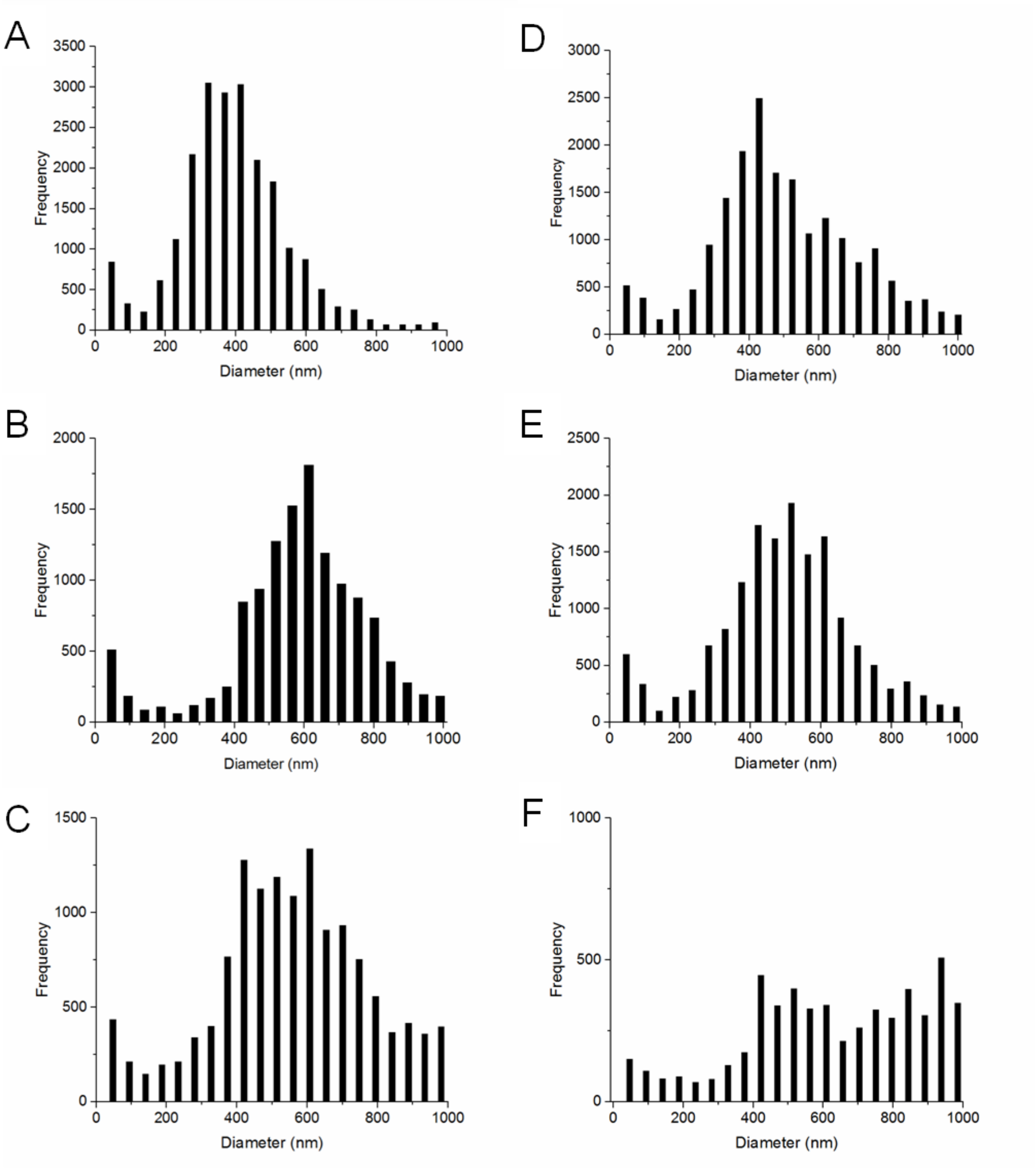

Figure S3: Histograms of the frequency as a function of the fibers diameter: P8N6 (A), P10N6 (B), P12N6 (C), P8N9 (D), P10N9 (E), P12N9 (F)

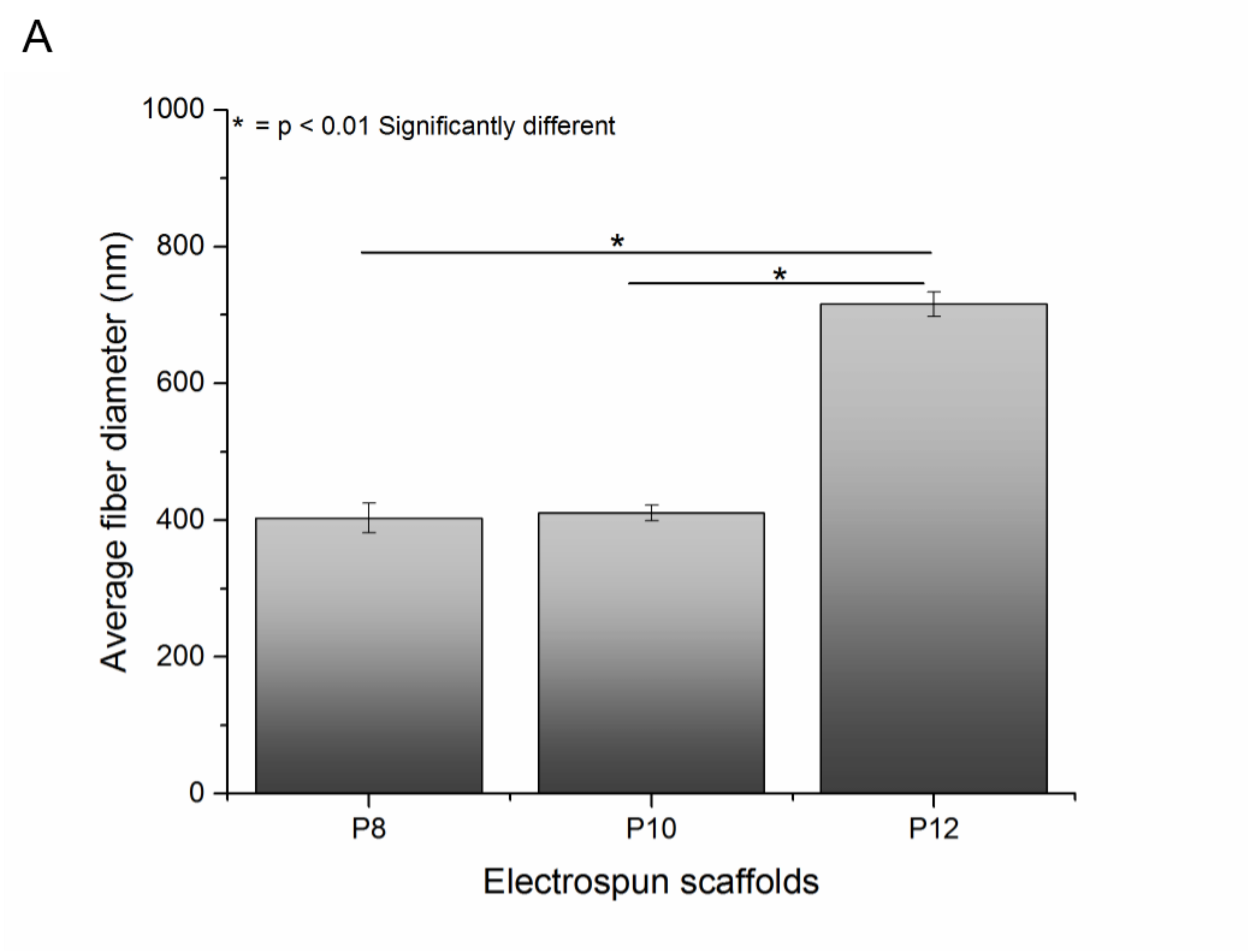

Figure S4 A: Fiber diameter histogram of electrospun scaffolds P8, P10, and P12. Error bars represent standard deviation.

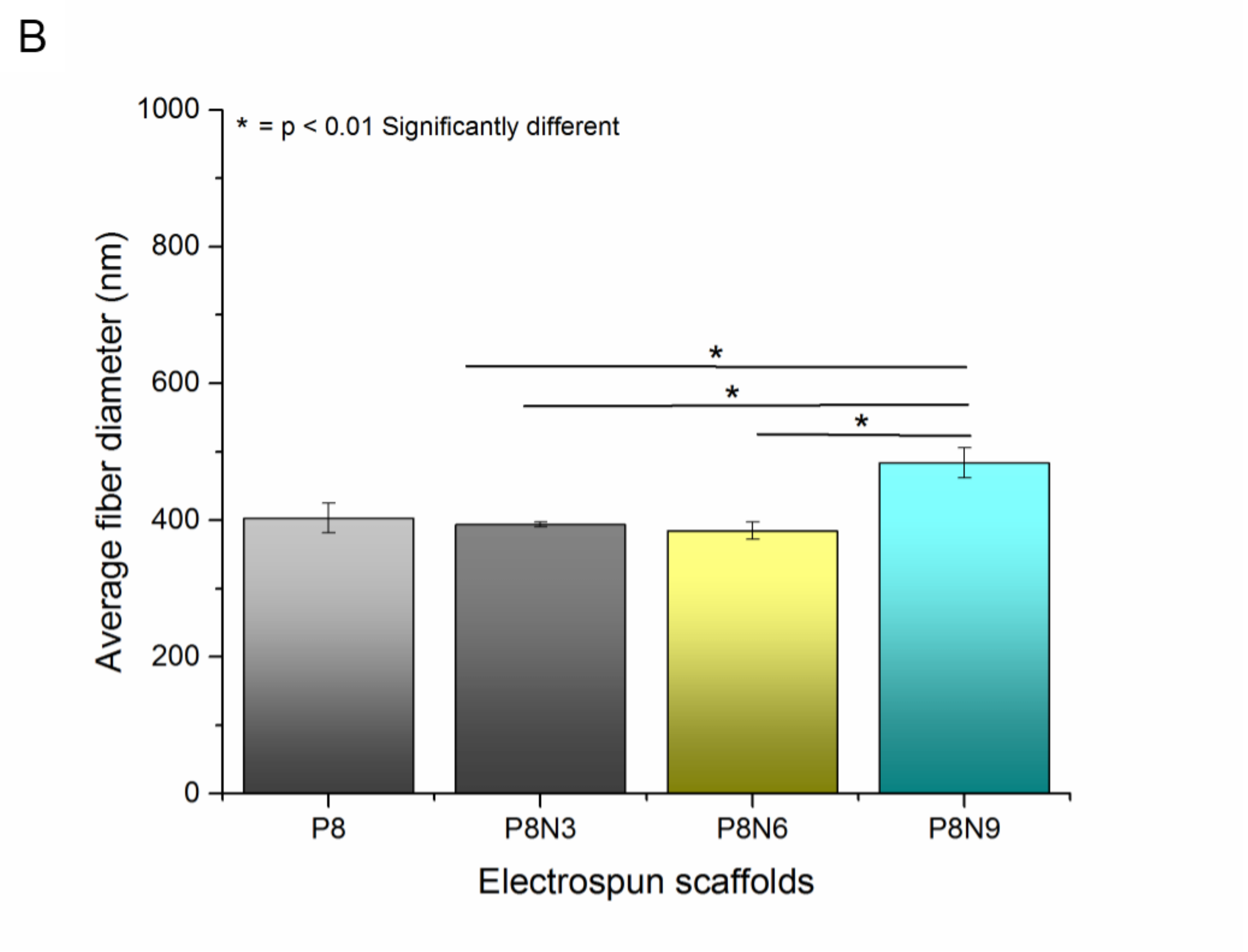

Figure S4 B: Fiber diameter histogram of electrospun scaffolds P8, P8N3, P8N6, P8N9. Error bars represent standard deviation.

C

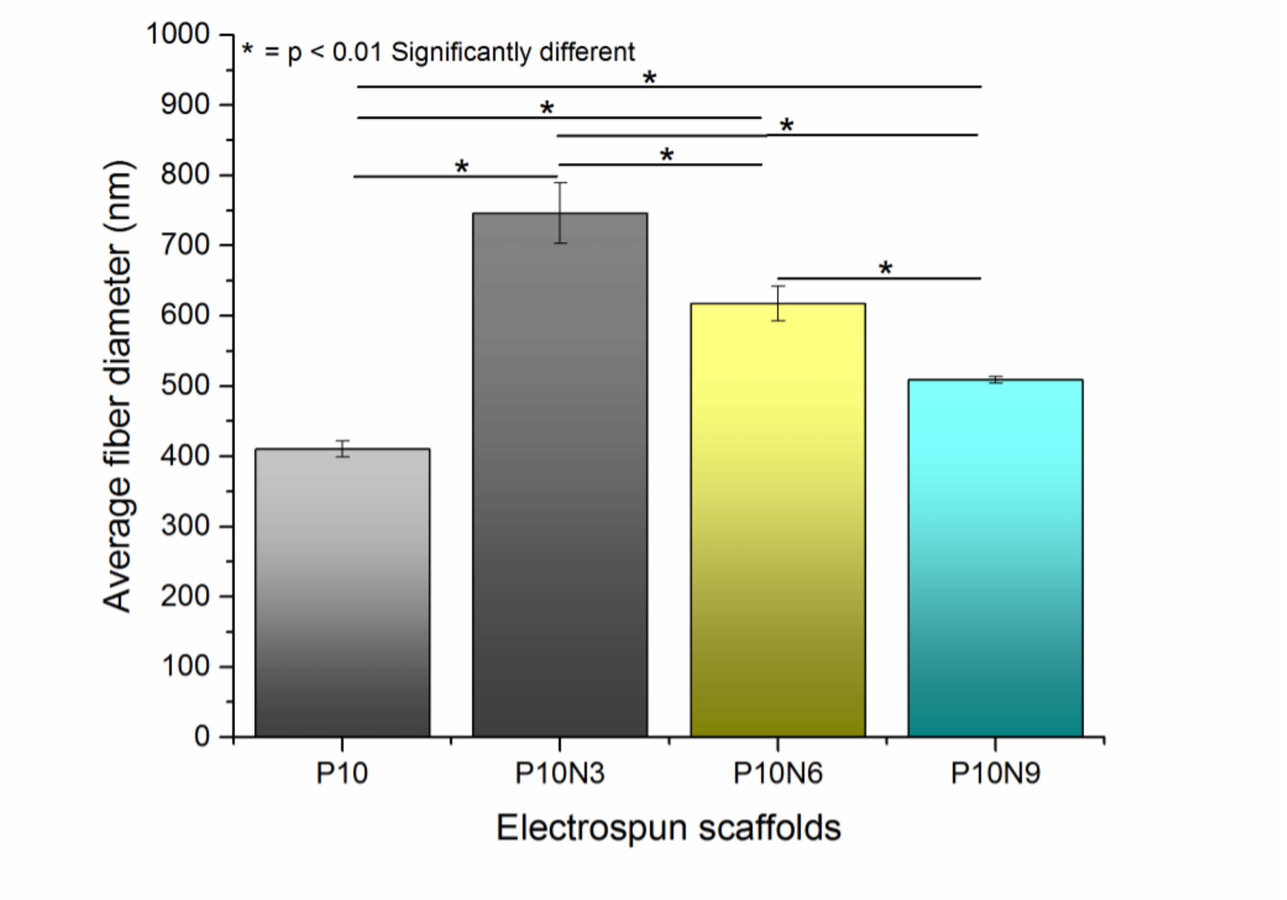

Figure S4 C: Fiber diameter histogram of electrospun scaffolds P10, P10N3, P10N6, P10N9. Error bars represent standard deviation.

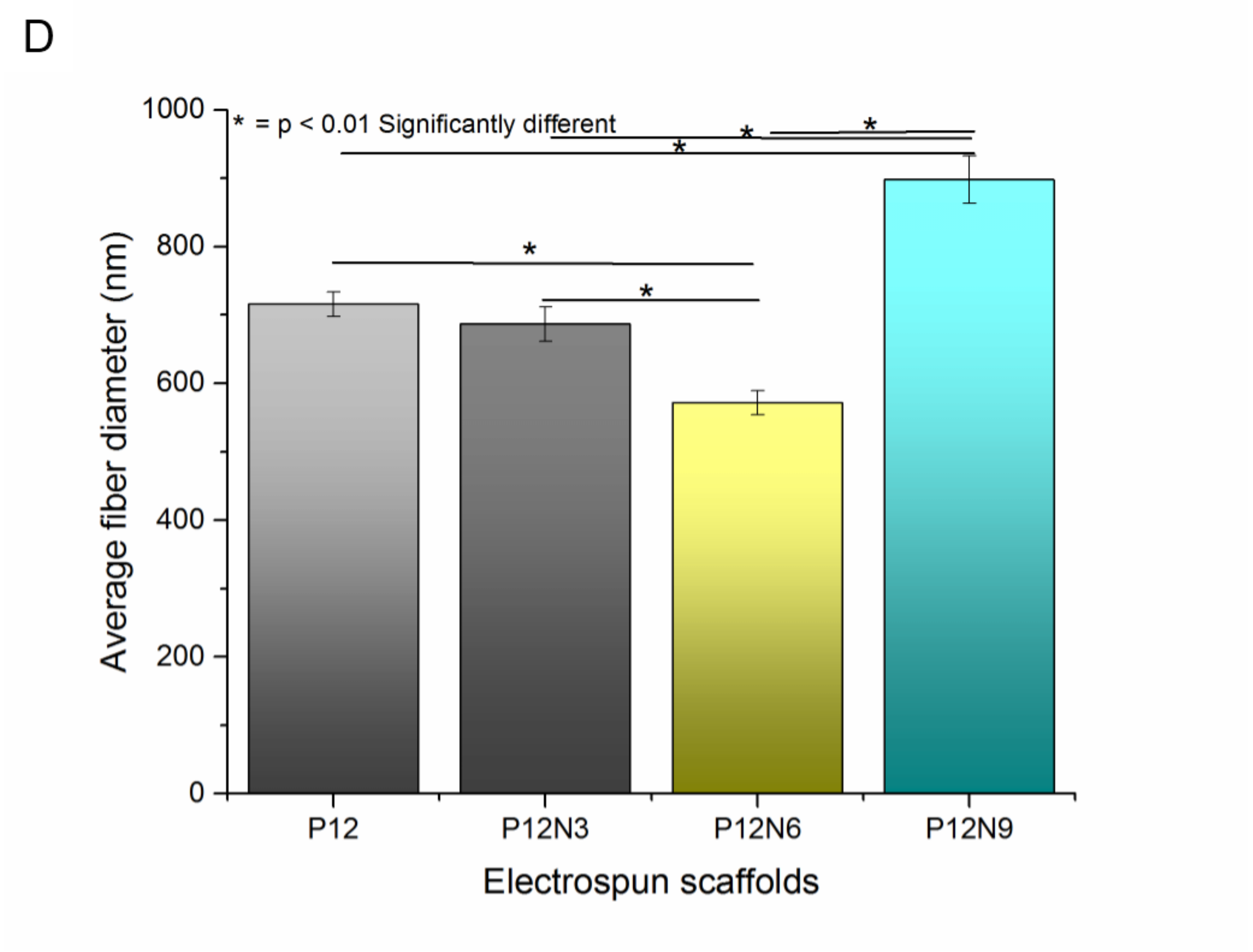

Figure S4 D: Fiber diameter histogram of electrospun scaffolds P12, P12N3, P12N6, P12N9. Error bars represent standard deviation.

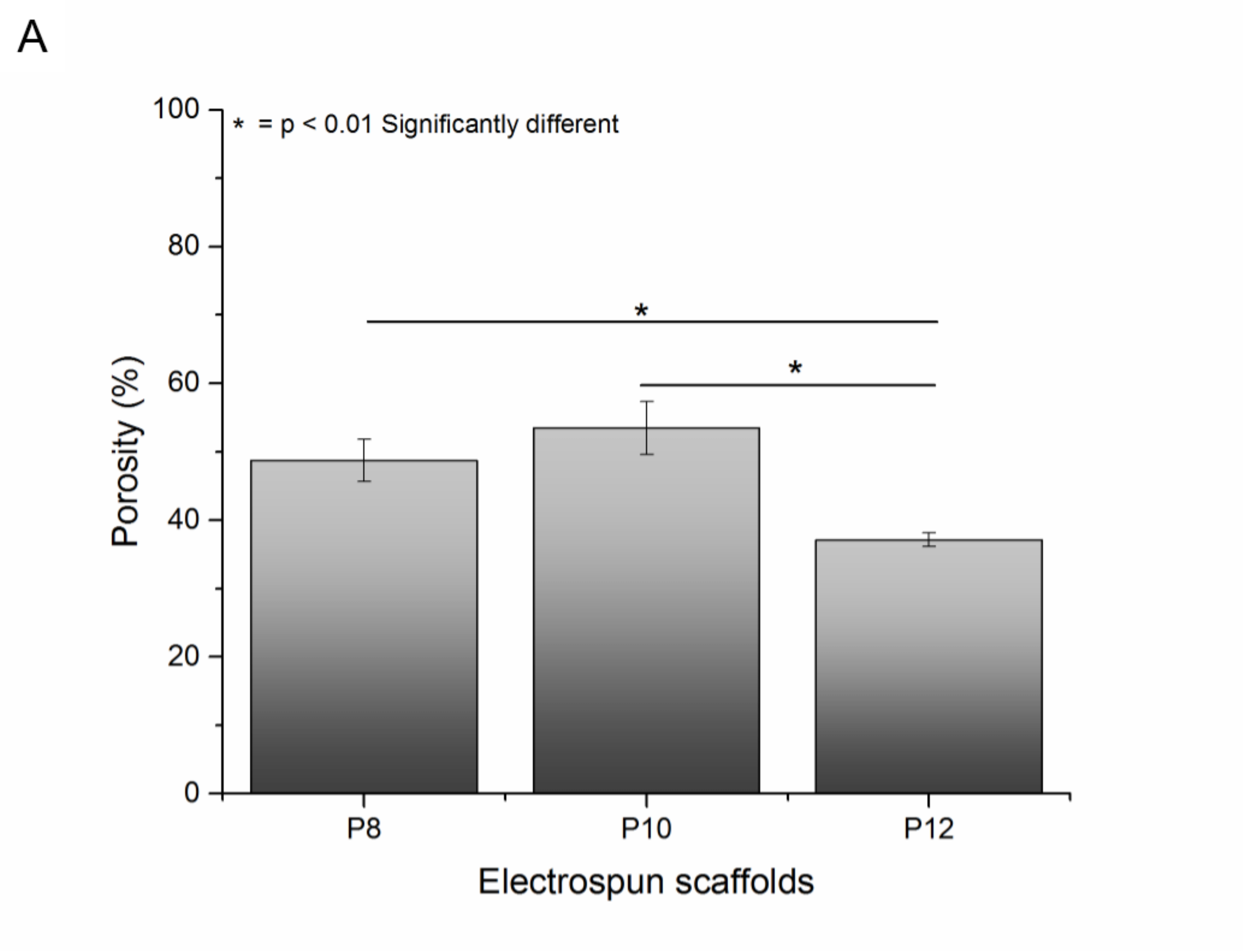

Figure S5 A: Porosity histogram of electrospun scaffolds P8, P10, P12. Error bars represent standard deviation.

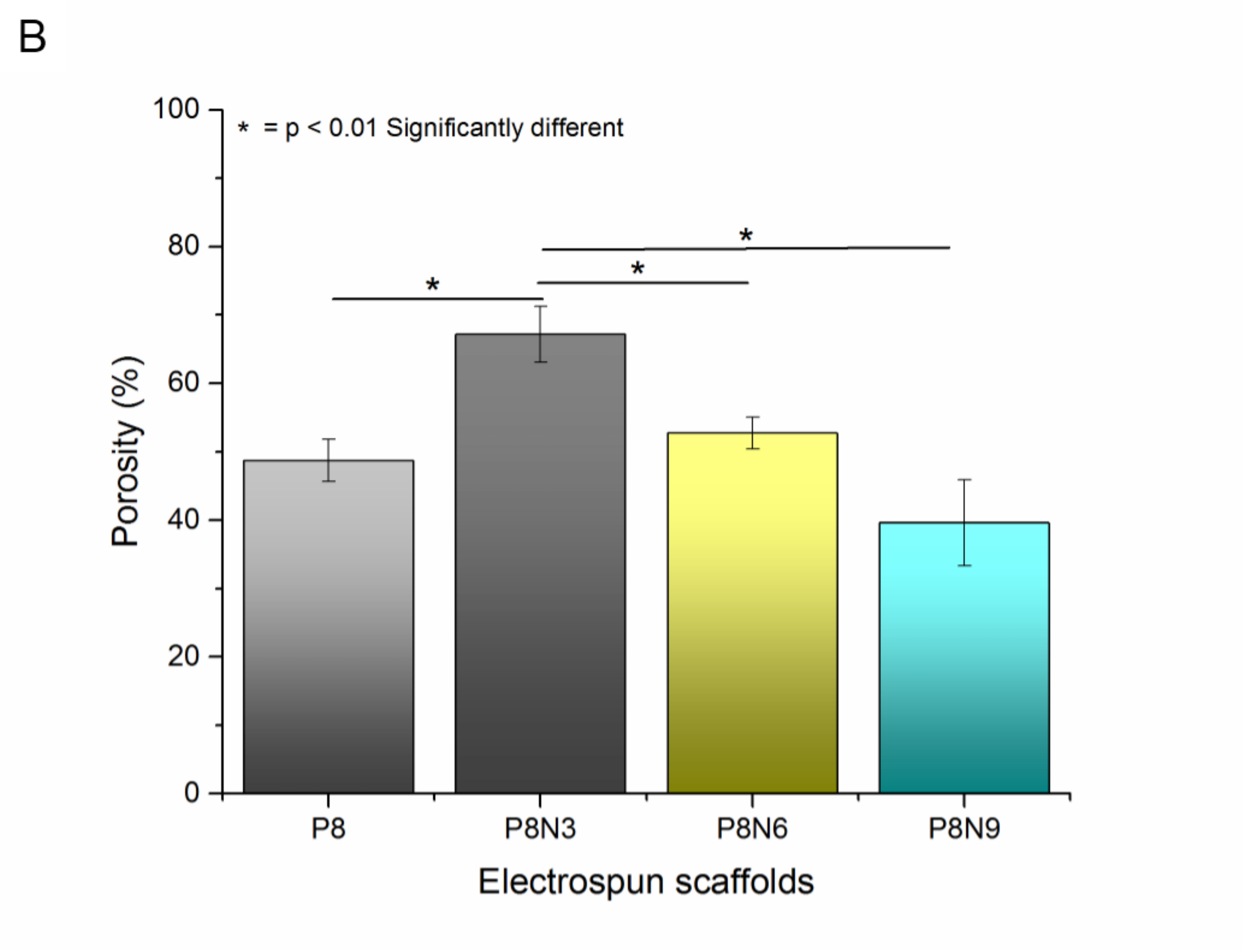

Figure S5 B: Porosity histogram of electrospun scaffolds P8, P8N3, P8N6, P8N9. Error bars represent standard deviation.

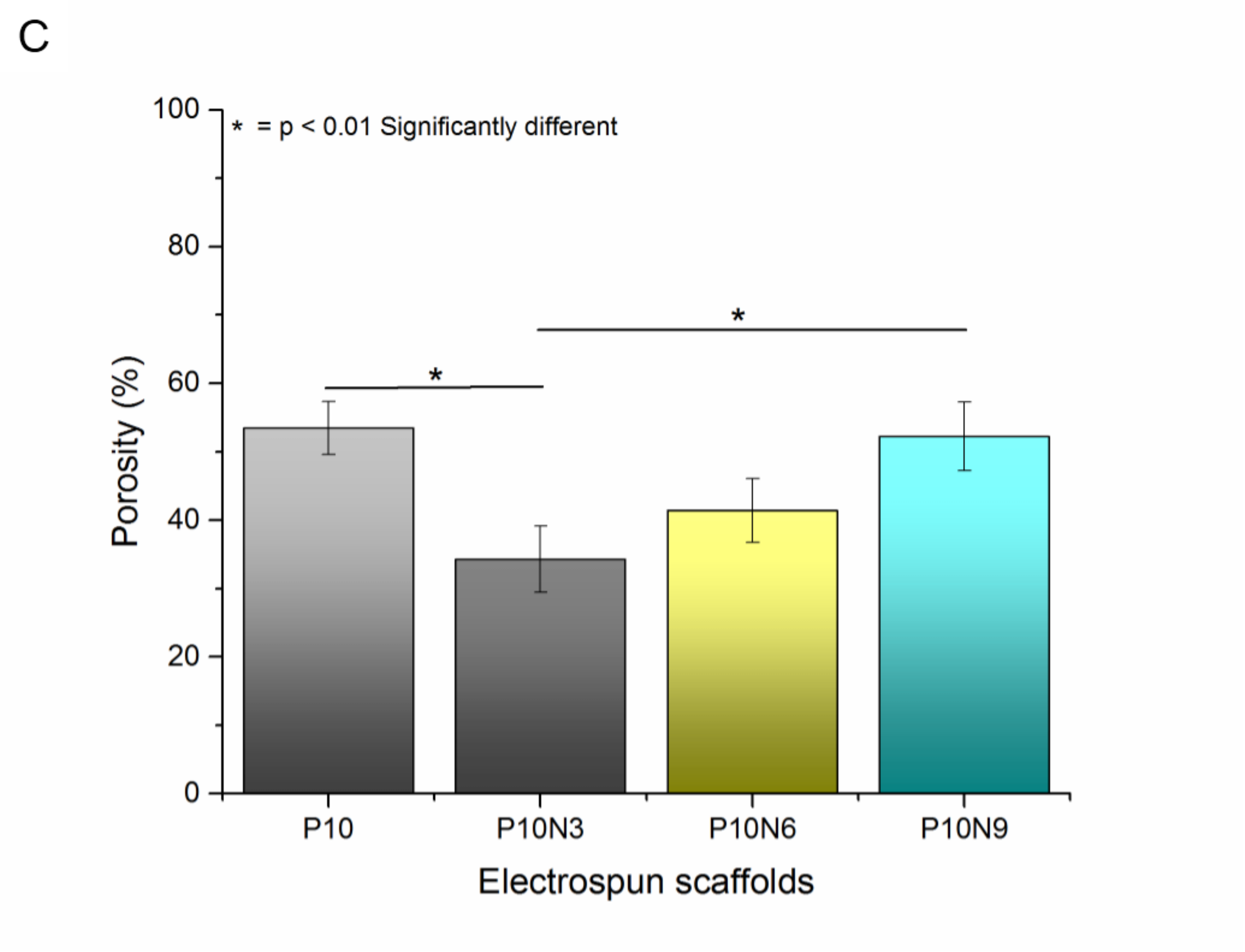

Figure S5 C: Porosity histogram of electrospun scaffolds P10, P10N3, P10N6, P10N9. Error bars represent standard deviation.

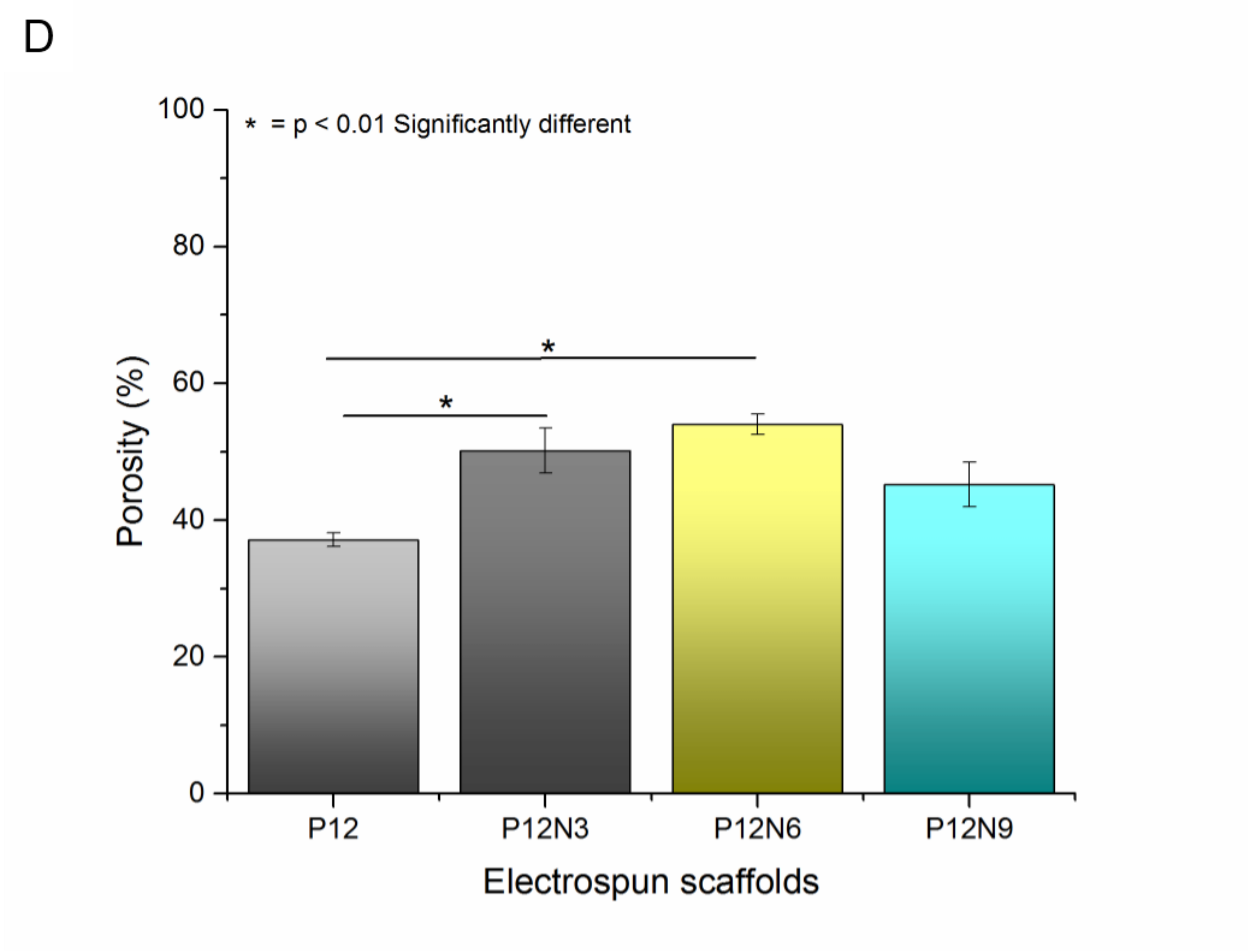

Figure S5 D: Porosity histogram of electrospun scaffolds P12, P12N3, P12N6, P12N9. Error bars represent standard deviation.

Table S1: Scaffolds morphology and wettability

Abbreviation	Mean fiber diameter [a] (nm)	Porosity (%)	Swelling (%) in 1M $LiPF_6$ EC:DMC 1:1 V/V solution
P8	402.93 ± 21.56	48.72 ± 3.07	n.a.[b]
P8N3	393.60 ± 3.65	67.19 ± 4.10	n.a.[b]
P8N6	384.73 ± 12.83	52.74 ± 2.35	n.a.[b]
P8N9	483.83 ± 21.72	39.61 ± 6.30	n.a.[b]
P10	410.37 ± 11.30	53.50 ± 3.89	201± 20
P10N3	746.03 ± 43.19	34.29 ± 4.89	270 ± 27
P10N6	617.93 ± 24.55	41.42 ± 4.69	336 ± 34
P10N9	508.97 ± 4.59	52.26 ± 5.01	1100 ± 110
P12	716.10 ± 18.06	37.11 ± 1.00	500 ± 50
P12N3	686.80 ± 25.12	50.18 ± 3.26	423 ± 42
P12N6	571.90 ± 17.66	53.99 ± 1.50	475 ± 48
P12N9	898.07 ± 34.42	45.19 ± 3.23	698 ± 70

[a] Mean value ± standard deviation (n > 1000 from three different images)
[b]n.a. = not available

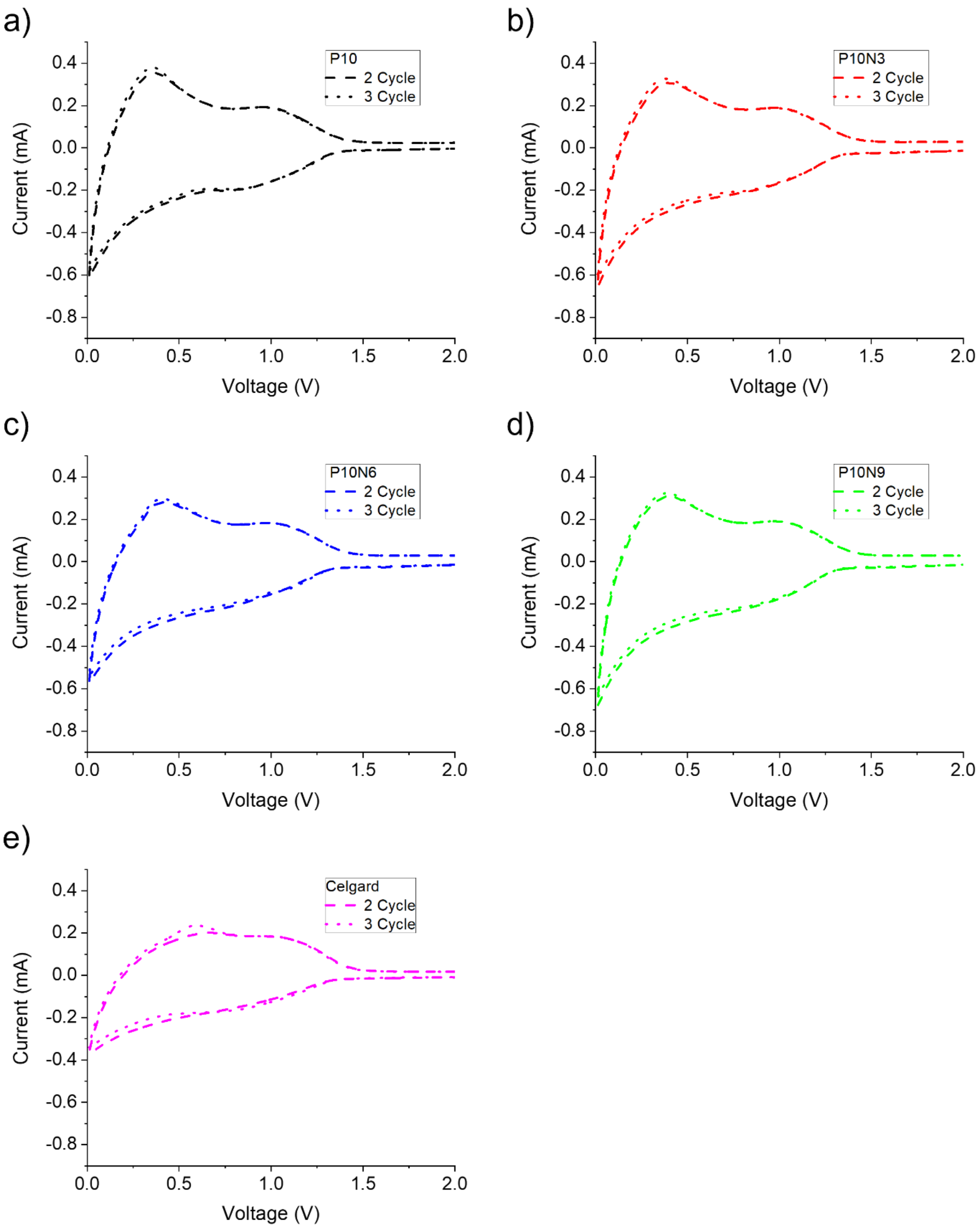

FigureS6: Ciclic Voltammteries of a) P10, b) P10N3, c) P10N6, d) P10N9 and e) Celgard.

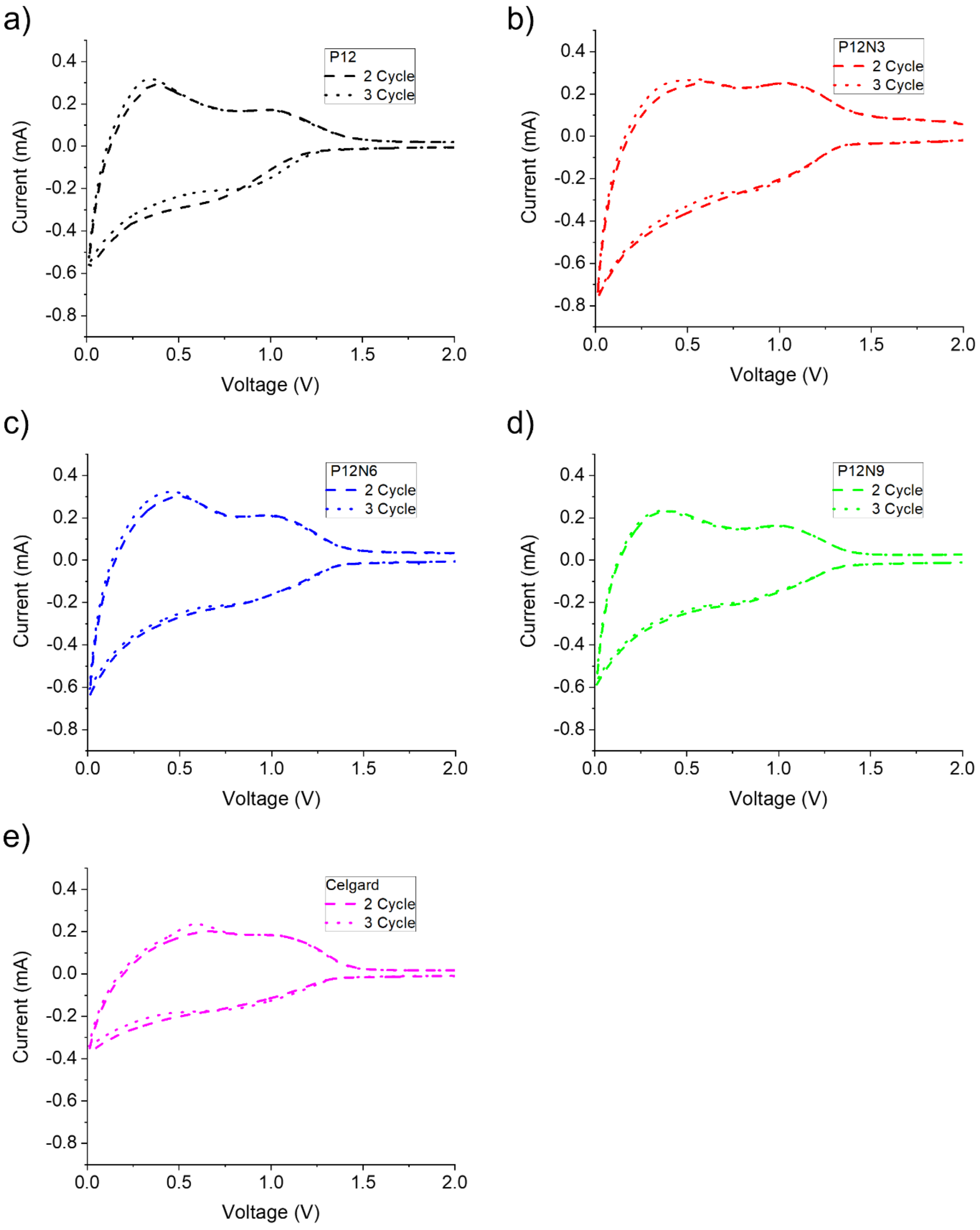

FigureS7: Ciclic Voltammetries of a) P12, b) P12N3, c) P12N6, d) P12N9 and e) Celgard.

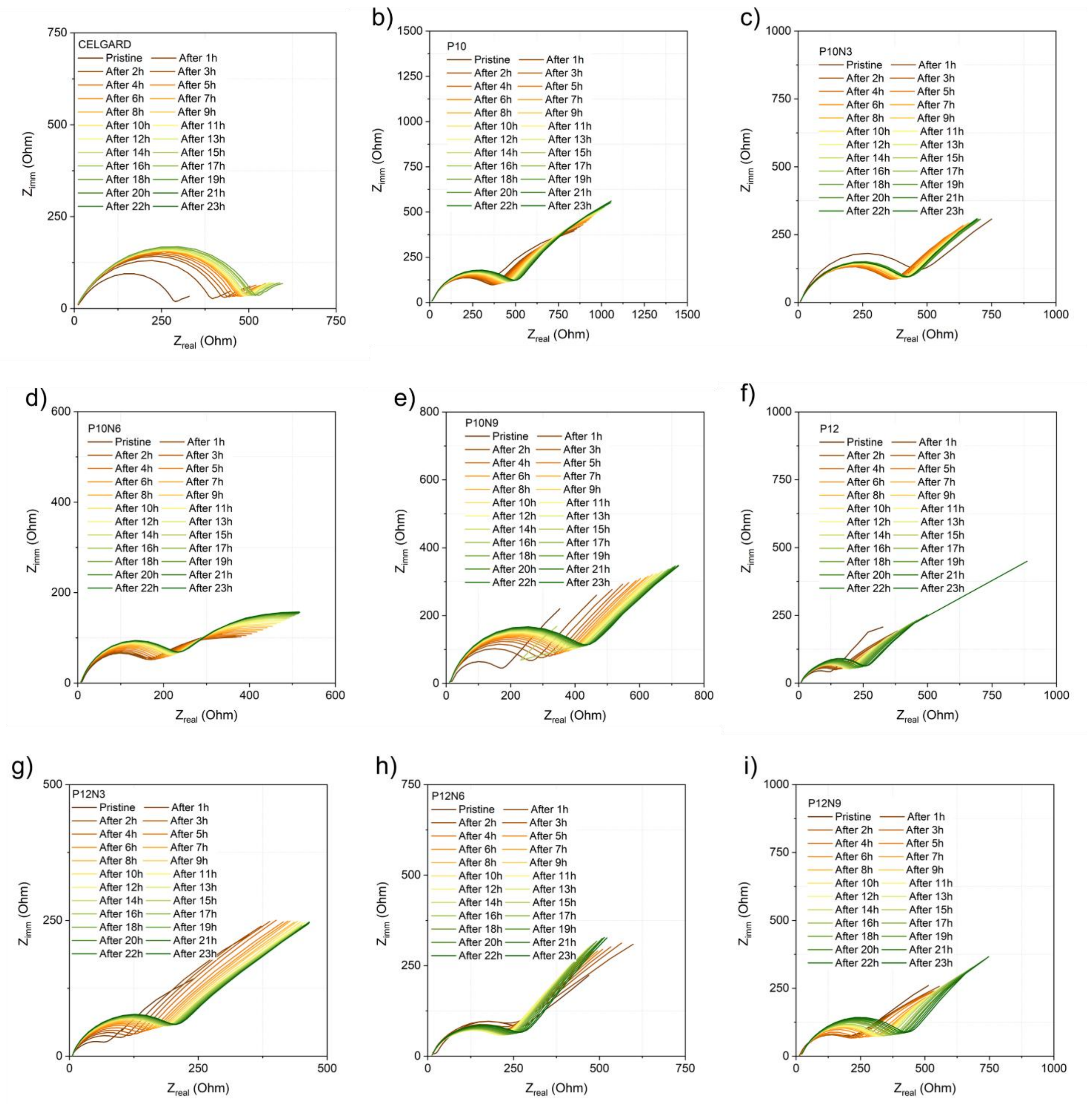

FigureS8: Impedance spectra obtained in Li/Li cells during 24h. Celgard was added as a comparison.
